# Recent Advances in Nanoporous Anodic Alumina: Principles, Engineering, and Applications

**DOI:** 10.3390/nano11020430

**Published:** 2021-02-08

**Authors:** Jakub T. Domagalski, Elisabet Xifre-Perez, Lluis F. Marsal

**Affiliations:** Departament d’Enginyeria Electrònica, Elèctrica i Automàtica, Universitat Rovira i Virgili, Avinguda dels Països Catalans, 26, 43007 Tarragona, Spain; jakub.domagalski@urv.cat (J.T.D.); elisabet.xifre@urv.cat (E.X.-P.)

**Keywords:** nanoporous anodic alumina, nanoengineering, nanostructures, surface chemistry, photonic crystals, sensors, templates, membranes, nanoparticles, nanocomposites

## Abstract

The development of aluminum anodization technology features many stages. With the story stretching for almost a century, rather straightforward—from current perspective—technology, raised into an iconic nanofabrication technique. The intrinsic properties of alumina porous structures constitute the vast utility in distinct fields. Nanoporous anodic alumina can be a starting point for: Templates, photonic structures, membranes, drug delivery platforms or nanoparticles, and more. Current state of the art would not be possible without decades of consecutive findings, during which, step by step, the technique was more understood. This review aims at providing an update regarding recent discoveries—improvements in the fabrication technology, a deeper understanding of the process, and a practical application of the material—providing a narrative supported with a proper background.

## 1. Introduction

In 2015, the nanotechnology worldwide market value amounted to $14.7 billion. Based on the growth rate observed back then, the market was predicted to grow by 375% and reach $55 billion in 2022 [1]. Three years before 2022—in 2019—market value of nanotechnology reached over $64 billion, exceeding the prediction significantly. The scale is even more impressive taking into account that so far, the majority of commercially available nanomaterials in the marked are currently at the initial stage of their product life cycle. What is more, since countries are often lacking precise regulations involving production, distribution, and use of nanomaterials, precautionary measures are applied that can potentially slow down the development [2]. For example, nanomaterials are excluded from the simplified authorization procedure [3]. We are still ahead of the rapid expansion to various branches of the industry [4]. Furthermore, this opinion is not alone. The European Committee proposed the term Key Enabling Technology (KET) defining the most promising technologies to secure strategic position and competitiveness of the European industry. Advanced materials, nanotechnology, nano and microelectronics, photonics, biotechnology, and advanced manufacturing have all been identified as KETs [5]. Although there are many materials/technologies that cover some of these aspects, only a few cover all of those mentioned. An example of such can be fabrication and utilization of nanoporous anodic alumina. 

Aluminum is the most frequently anodized metal. During the process, material is immersed in the electrolyte and the current flow is introduced to the system. The material of interest serves in such setup as an anode, hence the term anodization. With this approach, it is possible to grow a layer of an amorphous oxide on the material surface. Anodization is a process commonly used in the industry where it serves as a robust, cost-effective approach to provide the surface with better mechanical properties and higher chemical resistance of metals [6]. However, under carefully adjusted conditions, the created structure is highly ordered. In combination with intrinsic chemical and physical properties, material has been considered to various applications [7,8,9,10,11,12,13]. Amongst other materials, it offers fabrication simplicity and tailor-engineering versatility combined with the unprecedented self-ordered regularity as compared to other nanoporous materials [14,15]. What is more, the anodization process is significantly safer than the fabrication of another highly valued material—mesoporous silica—that involves highly toxic and dangerous hydrofluoric acid [16,17]. Fabrication of nanoporous anodic alumina is an industrially scalable and cost-effective process [18,19]. Taking into account the level of spatial ordering and the high regularity of the structure, nanoporous anodic alumina (NAA) can be considered as the most striking example amongst anodic metal oxides and, also, a mesoporous silica competitor. Contrary to soft and flexible aluminum, the created NAA layer is hard and brittle [20,21], although it has been demonstrated that thinner walls of the pores correlate positively with its ductility [22]. 

Since its first introduction to the industrial scale in 1923, fabrication technology has changed greatly. Observation of new discoveries and the continuous development is a fascinating demonstration of how science carves its path towards excellence. The precise nanotechnology tool nanofabrication of NAA began as a robust protective coating method—back then, no one was aware of its nanostructured morphology. Although this review will not provide elaborate details about the material’s fabrication development history, insight on particular milestones is provided. Instead, focus was laid on gathering the most crucial data about formation mechanism, its tailor-engineering, investigation of certain material properties, and the most recent applications. The aim was to provide a comprehensive review, convenient as a first step to work with nanoporous anodic alumina. 

## 2. Nanoporous Anodic Alumina (NAA): Definition and Formation Mechanism of the Porous Oxide

**Figure 1:**
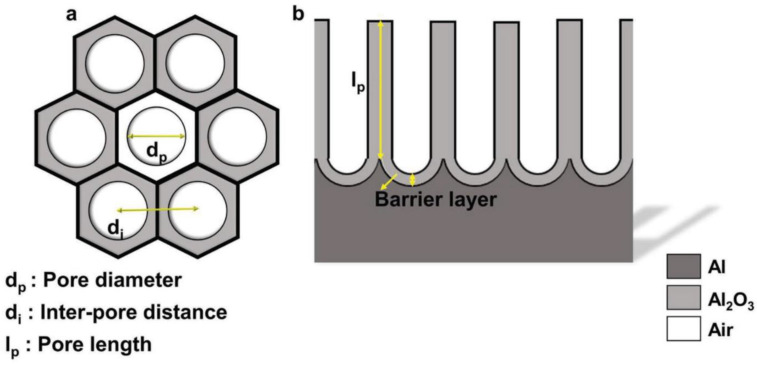
Schematic representation of nanoporous anodic alumina (NAA) structure. (**a**) Top-view with defined geometrical parameters; (**b**) cross-section with chemical composition. Reprinted with permission from [12]. Copyright 2017 Wiley.

NAA is formed by the electrochemical anodization of pure aluminum wafers and consists of a parallel array of pores surrounded by hexagonal cells of aluminum oxide (alumina). Each cell is in a direct contact with six others forming a structure that resembles a honeycomb. The pores grow in depth perpendicularly to the metallic surface as the anodization advances. A standard NAA structure can be defined with three physical parameters: The pore diameter (d_p_), the interpore distance (d_int_), and the length of the pores (l_p_). These geometrical features of NAA are shown in Figure 1 [12]. 

The pore diameter can range from 8 to 500 nm for NAA structures and depends on the anodization process conditions such as the applied potential, the electrolyte, and its temperature [23,24,25,26]. 

The interpore distance d_int_ indicates the average distance between the centers of two consecutive pores. This is an important value to determine the porosity of the structure since higher interpore distance translates to a smaller density of pores per surface unit. Higher concentration of the electrolyte and temperature seems to reduce the resulting interpore distance, while the applied potential was found to be in a positive correlation [27]. NAA presents high pore densities between 10^8^–10^11^ cm^−2^ and pore length ranging from a few nanometers up to millimeter scale [28]. 

The length of the pore l_p_ is proportional to the total current charge. During a standard anodization carried out under potentiostatic conditions, a brief initial rise and fall of the current precedes the stable plateau that enables to estimate thickness based on duration of the anodization [29,30]. Although the geometry of the pores remains constant during all the anodization process when the anodization parameters are kept invariant, it can be observed that pores tend to be wider close to the surface. It can be attributed to the longer exposure time, which causes slow oxide dissolution by the electrolyte. This may yield a certain degree of discrepancies as initial geometric features are determined by the process conditions. The dissolution rate of NAA in various acids is compared in the work of Poznyak et al. [31]. It was demonstrated to depend on the nature of the acid—the reactivity with respect to aluminum and the morphology of aluminum surface. 

The influence of conditions that enable the precise tailoring of NAA’s geometrical features such as the electrolyte, its temperature and additives, the anodization potential and current, and post-processing treatments are described in detail in the sections below. 

By chemical definition, NAA is an amorphous aluminum oxide with built up water and remains of the electrolyte ions incorporated during the anodization. The distribution of the impurities resembles layered onion-like structure with the highest concentration of ionic residues at the inner walls of the pores with a gradual decrease to the outside part of the pores [32,33,34]. This intake is an intrinsic factor partially responsible for the variations in the thermal conductivity of NAA reported in the literature. Recently, an attempt to systematize the observed effect was reported by Vera-Londono et al. [35]. It was established that thermal conductivity depends on the presence of ionic impurities—thus the electrolyte of choice—along with water built in the structure, and the crystalline form. As temperature increases to 100 °C, a drop in the thermal conductivity (~50%) is observed along with water loss. Later, however, continuous increase can be observed. Both removal of ionic impurities and transition into crystalline form result in higher thermal conductivity: 0.78 ± 0.19 W m^−1^ K^−1^ for the sulfuric NAA heated to 100 °C and reaching 4.16 ± 0.35 W m^−1^ K^−1^ when the sample was annealed at 1300 °C. 

NAA is an attractive and interesting material that presents important features. One of the most prominent features of NAA is its optical responsiveness that can be tailored to act as a photonic crystal [34,36]. It exhibits a high degree of transmittance in the visible light region [37,38]. Additionally, NAA has photoluminescent properties [39]. The origin of the photoluminescent properties of NAA was examined by Cantelli et al. [40]. Recombination centers derived from oxygen defects were proposed as the origin of the emission. The outlined hypothesis pointed onto different current densities present during the anodization in different electrolytes as the contributing factor to the quantity of oxygen vacancies inside the NAA. 

The material features also dielectric properties. The dielectric constant and loss are inversely proportional to the porosity of NAA and the applied alternating current frequency. The behavior of NAA is similar to other ceramic materials [41]. 

NAA is attractive not only due to its impressive enormous surface area combined with high chemical and thermal resistance, but due to the range of robust functionalization processes such as salinization [42], electrostatic interaction [43], and immune complexation [44] that allow versatile utility of such substrates: Sensors [45,46], templates [47], or drug delivery systems [48].

### 2.1. An Electrolytic Passivation of Aluminum 

**Figure 2:**
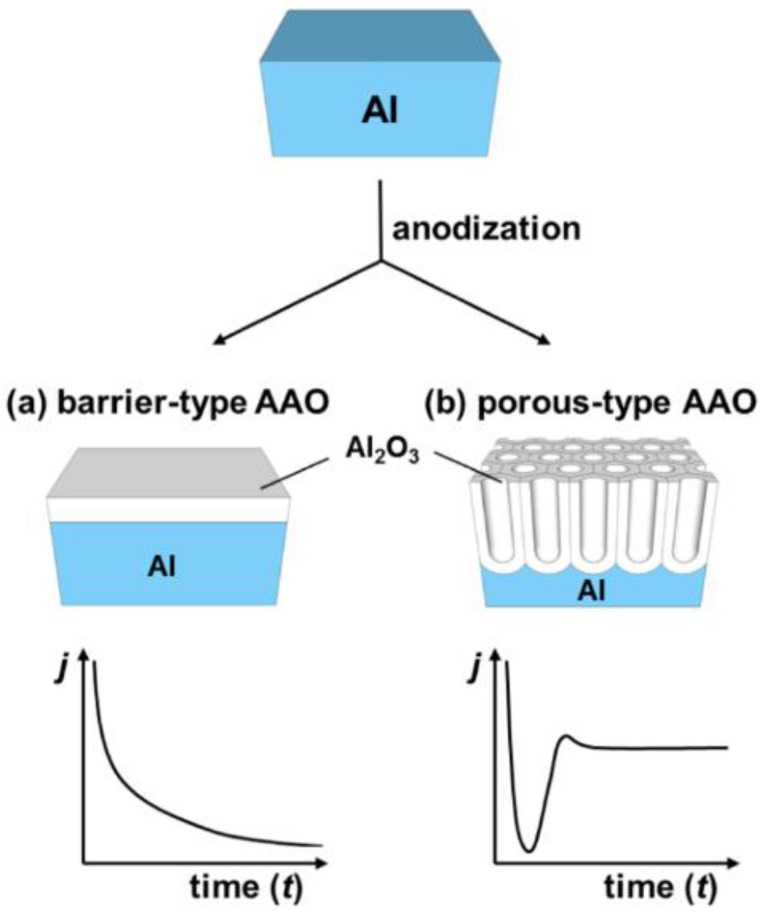
Types of anodic aluminum oxide and corresponding current-time transients. (**a**) Barrier type and (**b**) porous type. Reprinted with permission from [49]. Copyright 2014 American Chemical Society.

As mentioned before, NAA is obtained by the electrochemical etching of aluminum, however the electrochemical etching of aluminum does not always result in a porous structure of alumina: Different morphology of the grown oxide can be observed depending on the chemical character of the electrolyte. Electrolyte acidity is considered the major contribution factor to the distinct growth behavior. Anodization in a neutral electrolyte (borate, oxalate, citrate etc.; pH 5–7), that does not react with aluminum oxide yields a barrier-type anodic alumina as shown in Figure 2a [49]. However, an anodization performed in an acidic electrolyte (in which the oxide structure is slowly dissolved) results in the formation of a porous structure (Figure 2b) [50,51]. The most common electrolytes applied to create porous alumina are phosphorous, oxalic, and sulfuric acids—all featuring unique current/voltage parameters and structure geometry [52]. The anodization profile reflects the formation stage of the structure—the creation of a barrier layer and the growth of pores. In potentiostatic conditions, the formation of a barrier-type oxide follows exponential decrease of the current over time that goes along with the growth rate decrease. This retardation of the current flow is also reflected in a significant decrease of accessible oxide thickness as compared to the porous structure (Figure 2a). The formation of a nanoporous anodic alumina can be followed along with the current flow changes. Initial oscillations are related to the rearrangement of the structure with succeeding stabilization, when reaction reaches equilibrium. Then, quasi-stable current flow can be maintained for up to several days providing a steady growth rate (Figure 2b). A nanoporous film can reach even several hundreds of micrometers with a thickness linearly dependent on the applied current charge [24].

Through the radiotracer studies, it was possible to determine the exact place in which the formation of the oxide occurs in both scenarios. During the formation of a barrier oxide, growth occurs simultaneously at two interfaces: Oxide/electrolyte and metal/oxide. When a porous type alumina is formed, growth takes place only at the metal/oxide interface [53,54]. In further parts of the review, only porous structures will be discussed due to their unique, complex morphology in the micro- and nanoscale. 

### 2.2. Pore Growth Mechanism and Spatial Ordering

**Figure 3:**
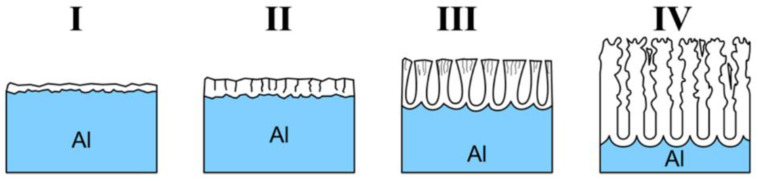
Scheme of step-by-step formation of porous oxide. (**I**) Formation of barrier layer, (**II**) formation of local cavities, (**III**) growth of porous structure, and (**IV**) schematic of nanoporous structure with initially disordered structure and following self-ordering. Reprinted (adapted) with permission from [49]. Copyright 2014 American Chemical Society.

While the word ‘formation’ may intuitively point to the one-way character of the process, it is in fact a result of several reactions occurring collaterally [55]. Pore formation during the anodization of aluminum is generally considered as a consequence of the equilibrium between two opposing changes in the structure: (i) Growth of the aluminum oxide at the metal/oxide interface and (ii) dissolution of the aluminum oxide at the oxide/electrolyte interface [56]. Simplified representation of the changes that occur during the anodization are visualized in Figure 3. When a constant anodic potential is applied, the entire surface of aluminum gets covered with a thin oxide barrier layer (I, Figure 3). As the process continues, electric resistance of the setup gradually increases along with the oxide layer growth resulting in the drop of the current flow, until it reaches a minimum value (II, Figure 3). O’Sullivan and Wood [27] suggested that at this stage the electric field concentrates on the local imperfections in the barrier layer. Different explanation, focusing on the local cracking in the barrier layer that facilitates the electrolyte penetration, was proposed by Thompson [57,58]. Polarization of Al-O bonds that goes along with the increased electric field can further promote the dissolution of the oxide. During this stage, a rapid increase of the current density can be observed (III, Figure 3) following a slight decrease and stabilization (IV, Figure 3). This slight drop has been related with a decrease in the pore density at the beginning of the process attributed to merging of pores. 

The growth of highly organized structure from the very beginning of the process is possible only after certain preparations or pre-patterning. As shown in Figure 3, initial porous structure is disordered. However, as the process continues, the structure below becomes ordered. To avoid the presence of disordered pores at the outer surface, the golden standard utilized during NAA fabrication is the two-step anodization process [17]. Once the structure becomes regular during the first anodization step, the oxide layer is selectively removed—surface of the aluminum plate features cavities that mirrors geometry of the organized structure. Then, when the second step of anodization is applied, the growth immediately follows this geometry. It is important to note that high regularity can be obtained only under narrow sets of anodization conditions. There are many factors that affect the course of the anodization process: Potential, current flow, ion migration, local depletion, and in certain conditions, even electrical breakdown.

The extended investigation of the anodic alumina formation had led to the conclusion that growth and self-organization of alumina are significantly affected by the internal stress occurring during the formation process. A volume expansion is one of the crucial factors that have an impact on self-ordering. It was discovered [59] that the highest ordering occurs with a moderate volume expansion. Either the significant volume expansion or the contraction is accompanied by more disordered pores. Evolution of the internal stress that occurs during pores initiation and growth leads to the increase of the compressive stress with raising alumina thickness [60]. What is more, the extent of volume expansion can be linked to the regularity of the obtained structure. A simple way to quantify the volume expansion is by comparison between the final NAA volume to the initial thickness of aluminum. The relation between the spatial ordering and the observed volume expansion is investigated in the work of Jessensky [59]. Either contraction or higher expansion would result in a lower degree of ordering. These results were independently supported in the research of Nielsh et al. [61]. They proposed the 10% porosity rule as a requirement for the highest spatial ordering, which translates to the volume expansion of 1.23 independently of the anodization conditions. While in the most cases reported results remain in a good agreement with this implication, there are some exceptions. For example, it was possible to obtain highly ordered structures with 0.3 M selenic acid at much lower porosity of 0.8% [23].

Completely ordered NAA structures can be obtained for certain electrolytes under a narrow range of anodization conditions that are discussed in the next section. 

### 2.3. Electrolyte Specific NAA Geometry

While development of the technology to increase the control over the process and reach high regularity is still an important research objective, decades ago its progress was significantly restricted by the existence of the initial, disordered layer of the alumina combined with a lack of sufficient tools to force the high ordering from the very beginning of the oxide growth. To date, the most frequently cited article involving nanoporous anodic alumina—the one that elevated the moderately attractive surface functionalization approach into a sophisticated nanotechnology tool—was the discovery done by Masuda and Fukuda [17]. The exponential growth of publications in two recent decades that followed the discovery is justified by the impressive regularity achievable with this new approach [62,63]. Properties of the grown NAA depend highly on the applied potential (with the resulting current) and a specific electrolyte. An initially disordered porous structure starts to form a regular array of hexagonal cells. The size of these cells is linearly dependent on the applied potential. However, it was observed that the highly regular morphology featuring low quantity of structural defects can be achieved only in narrow sets of conditions [64,65]. Each electrolyte utilized in the formation of nanoporous anodic alumina features a set of conditions: Potential, electrolyte concentration, and temperature in which high ordering can be achieved. 

It was empirically established in many independent experiments, that interpore distance—or “cell size”—of the NAA structure depends directly on the applied voltage being defined as:

D_int_ = k U_an_ (1)

where U_an_ is an anodization voltage and k the constant that can be roughly estimated as k = 2.5 nm V^−1^, independently of the applied electrolyte [49,66]. However, self-ordered growth occurs only in a narrow range of anodization potential that is unique to particular ionic species. An incentive to seek for new electrolytes and self-ordering regimes is justified by the perspective to provide better covering of already accessible geometry [67]. 

**Figure 4:**
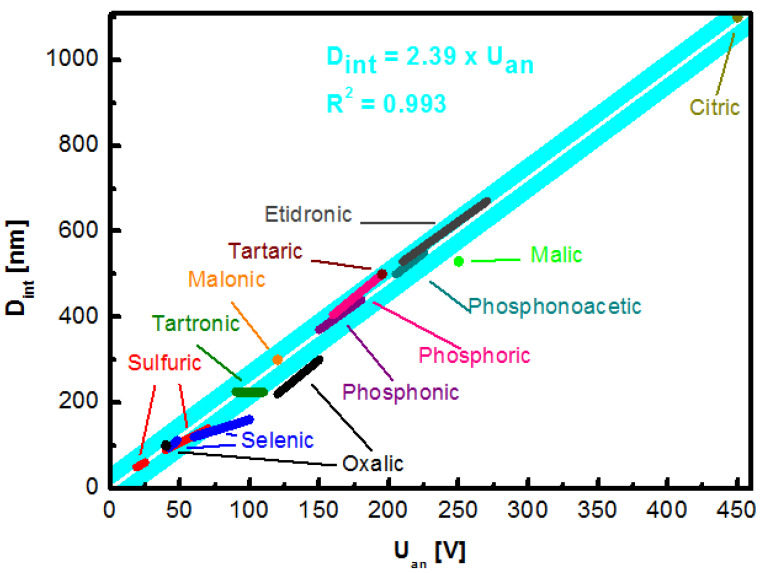
Linear relationship between anodization potential and interpore distance for self-ordered NAA formed during anodization in various electrolytes: Sulfuric, selenic, oxalic, tartronic, malonic, phosphonic, phosphoric, tartaric, phosphonoacetic, etidronic, malic, and citric acids.

A complete revision of the electrolytes reported in the literature that provide a high regularity of the porous structure and the corresponding range of obtainable pore distance is gathered in Table 1 and graphically represented in Figure 4. The value of k constant was based on calculation of all the values available in reports included in the Table 1. The presented value of k = 2.39 nm V^−1^ is close to the previously proposed simplified estimation [49,66].

The majority of research articles involves the fabrication of NAA grown in one of three acids considered “standard”: Sulfuric acid (H_2_SO_4_), oxalic (H_2_C_2_O_4_), and phosphoric (H_3_PO_4_) [27,52]. They are well-known and have been utilized for decades. Sulfuric acid was the first electrolyte used to yield an anodic alumina layer—initially with the intention to improve the corrosion resistance and hardness of the aluminum components. Amongst the aforementioned, it provides a triangular lattice with the highest density, which is a desirable feature in microelectronics [68,69]. What is more, the sulfuric-NAA provides the highest versatility in terms of an available cell size. Additionally, structures formed during the hard anodization exhibit high hardness of up to 400 Hv, attractive in terms of mechanical performance [70]. Even more impressive physical properties can be achieved with the novel discovery of the etidronic-NAA. The structures made with etidronic acid are 50% harder than with the sulfuric acid hard anodization (610 Hv) and can be further strengthened with thermal annealing (769 Hv) while featuring a low porosity of ~4%. What is more, the geometry of etidronic-NAA with near-subwavelength periodicity results in a significant reflection of light in the visible region: 490—760 nm [71]. Another phosphonate compound explored as an electrolyte for aluminum anodization is phosphonoacetic acid however, it has not been thoroughly investigated so far [72]. Different instance of a structure highly attractive in terms of optical properties is oxalic acid. The oxalic-NAA is often used to prepare photonic structures [73,74]. Fabrication of photonic crystals with improved color saturation preserving the aluminum substrate, which is possible due to introduction of short, high-voltage (250 V) anodization step subsequent to the conventional sinusoidal pulse anodization, was demonstrated by Sun et al. [75]. The oxalic-NAA is also one of the most prominent examples with regards to the photoluminescence performance, exceeding that of the sulfuric- and the phosphoric-NAA and can be adjusted with fabrication conditions [76,77]. Another example of a photoluminescence-active structure can be the arsenic-NAA [78]. This recent approach is characterized with a structure featuring much thicker skeleton of pure alumina as compared to other typical electrolytes. Moreover, the arsenic-NAA exhibits unique white photoluminescence emission (515 nm) under UV irradiation (254 nm). On the other hand, the tartaric-NAA features a broad spectrum of blue luminescence (400–750 nm). Detailed analysis of the as-prepared and annealed tartaric-NAA films revealed two sources of the emission [79]. The highest intensity peak with a maximum at 460 nm originated from bulk and adsorbed OH groups, while amorphous carbon derived from the electrolyte contributed to the peak at 550 nm. Practically, the tartaric acid-NAA remains unexplored with regards to nanotechnology, but the mixture of tartaric-sulfuric acid has been implemented as an alternative replacing chromic acid anodization for European aeronautical industry in 2014 [80]. Recently, a new electrolyte has been found to yield highly ordered porous structure. A high degree of self-ordering with malic acid was discovery by Zajączkowska et al. [81]. Additionally, it was observed that the repetition of anodization—applying the same external anodization parameters—affects the current-time transient. It was suggested that a high degree of malate ion incorporated into a NAA structure could promote attraction of these ions to the Al electrode further facilitating formation of the oxide structure—the first such observation reported in the literature so far. 

Another acid that has been explored moderately recently (reported by Nishinaga et al. [23]) that can be used to yield a porous structure is selenic acid. The great potential of the selenic-NAA stems from its low porosity, colorlessness, and high transparency. Intriguingly, as compared to the NAA formed with organic electrolyte, it does not exhibit photoluminescence. It is also competitive with regards to the sulfuric-NAA due to rapid formation of the self-ordered structure of 10-nm pore diameter within 1 h. Similarl to sulfuric acid, self-ordering can be achieved for more than one potential range providing for higher fabrication versatility [23,82,83].

**Table 1:** Interpore distance reported for nanoporous anodic alumina anodized in various electrolytes.

Interpore Distance [nm]	Electrolyte	Concentration	Applied Potential [V]	Temperature [°C]	Reference
50–60	Sulfuric acid	0.3 M	19–25	5	[52]
90–140	Sulfuric acid	10 wt %	40–70	0.1	[84]
95–112	Selenic acid	0.3 M	42–48	20	[23,85]
100	Oxalic acid	0.3 M	40	5	[52]
120–160	Selenic acid	0.3 M	60–100	0	[82]
225	Tartronic acid	0.3 M	90–110	0.5	[86]
220–300	Oxalic acid	0.3 M	120–150	1–2	[24]
300	Malonic acid	5.0 M	120	0–1	[87,88]
370–440	Phosphonic acid	0.5–2.0 M	150–180	0–20	[89]
405–500	Phosphoric acid	0.3 M	160–195	5	[52]
500	Tartaric acid	2–4 wt %	195	5	[90]
530	Malic acid	0.5 M	230	5	[81]
500–550	Phosphonoacetic acid	0.1–0.9 M	205–225	10	[72]
530–670	Etidronic acid	0.3 M	210–270	0–40	[66,91]
1100	Citric acid	0.1–1 M	260–450	10–30	[92]

#### Impact of Temperature and Additives

Temperature of the electrolyte during anodization is an important factor affecting the formation of NAA. Higher temperature is a convenient measure to accelerate the alumina growth and adjust the resultant pore diameter not affecting interpore distance simultaneously [93,94]. Evaluation how—independently of the electrolyte temperature—temperature of the aluminum anode can impact the formation of NAA was performed by Chernyakova et al. [95]. For the temperature increase between 5 °C and 60 °C d_p_ and d_int_ remain unchanged, while structural ordering has increased. Results indicate that the rate of the NAA chemical dissolution is not temperature dependent. Furthermore, above 60 °C the self-ordering drops and formed pores are 1.7 times broader. However, outcome of the anodization can be also altered using addition of various chemicals to the electrolyte. 

**Figure 5:**
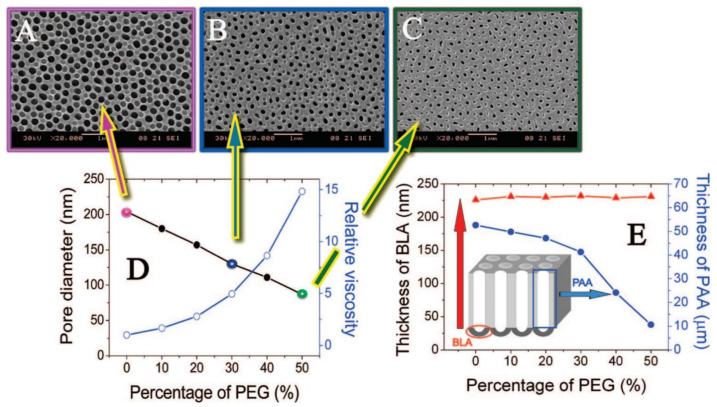
Impact of poly(ethylene glycol) (PEG) concentration on morphology of formed NAA films. Concentration of PEG in the electrolyte: (**A**) 0%, (**B**) 30%, (**C**) 50%, (**D**) influence of PEG content on the pore diameter and (**E**) the NAA film and barrier layer thickness in relation to PEG content in the electrolyte. Reprinted with permission from [103]. Copyright 2008 American Chemical Society.

Ethanol is a common electrolyte additive that enables to perform anodization below 0 °C reaching lower current densities. It allows to yield even smaller pores, for example 8 nm diameter pores for the sulfuric-NAA [26,96,97]. It was recently demonstrated that addition of ethanol improves the formation of NAA in sulfuric acid, suppressing its chemical dissolution and the anodization rate. What is more, the anodization was possible even with 50% ethanol content in the electrolyte [98]. Another common additive is ethylene glycol. It increases viscosity of the electrolyte decreasing the dissociation constant. Consequently, reduced electrolyte conductivity decreases current density. It is especially useful when anodizing low-purity aluminum as the increase of the current density may occur due to the localized impurities. Additionally, the anodization in a broader range of potential is possible without burning phenomenon. Yet, above certain critical potential values, hillocks and cracks occur decreasing the quality of resulting NAA [99]. The presence of ethylene glycol in the electrolyte for aluminum anodization facilitates oxidation of the intermetallic phase. Consequently, the connection between adjacent cells is weakened and voids form at the three cell junction—the effect intensifies along with raising ethylene glycol content [100]. Furthermore, intensified oxidation was linked with the increased incorporation of elements that does not originate from the electrolyte. It was established that increased intake derives from the elements present in the AA7075 alloy. Additionally, the growth rate had decreased as compared to the anodization without ethylene glycol [101]. When different alcohols are compared, effects are more pronounced for polyhydric alcohols [102]. However, not every aspect of electrolyte additives has been revealed so far. The challenge is their impact seems to be independently affected by other modifiers such as electrolyte, current density, pH, viscosity, etc. Anodization with addition of poly(ethylene glycol) (PEG) enables to alter pore diameter of the grown structure independently of the anodization voltage [103]. Authors attribute this behavior to the increase of the electric field strength (PEG has lower dielectric coefficient than water) and restricted chemical dissolution process. It was observed that immersion of the NAA films in the acid solution with 50% addition of PEG resulted in 4 times slower dissolution as compared to solution without PEG. As a result, it was possible to modulate the pore diameter of the structure using different content of PEG, while other parameters (acid concentration, anodization voltage, and electrolyte temperature) remained constant. The impact of PEG containing electrolyte on the morphology of NAA is shown in Figure 5. 

Alternatively, an electrolyte additive may promote the incorporation of elements meant to improve properties of alumina matrix. The addition of lithium phosphate to the electrolyte successfully incorporating lithium ions into NAA during its formation is presented in [104]. The incorporation of metal ions may be potentially attractive with the intention to increase the conductivity of the material [105]. The interesting experiment involving the electrolyte composition has been presented by Christoulaki et al. [106]. Water in sulfuric and oxalic acid has been replaced with deuterated water, and in one case, the electrolyte composed of D_2_SO_4_ in D_2_O was used. Use of deuterated water resulted in the 20% reduction of the pore diameter, improved pore ordering and increased growth rate. Observed behavior has been attributed to the decrease of the alumina formation activation energy. Furthermore, such electrolytes enabled to analyze the incorporation of hydroxyl groups during the NAA formation and effects of prolonged immersion in the solvent. Small-Angle Neutron Scattering shown no significant difference in incorporation rate between H_2_O and D_2_O pointing on the weak OH incorporation, while the hydration through immersion shown to be a slow process. 

### 2.4. Mild and Hard Anodization: Two Growth Regimes

For a long time, the default approach to the anodization of aluminum for the purpose of nanotechnology and research was the one performed under the moderate and constant potential—individually adjusted to each electrolyte—between the electrodes; under which the current flow is determined by reactions’ equilibrium. Due to the constant potential and the low current flow (that is typically below 30 mA cm^−2^) such a process is called potentiostatic mild anodization (MA). MA conditions result in a predictable course of the process and the stable growth rate of 1–3 µm h^−1^. However, several restrains of the process such as limited growth rate (the fabrication of self-standing membrane may require days of anodization), encouraged the exploration for a more practical, fast approach. What is more, self-ordering of NAA have narrow windows and discovery of new ordering regimes became the quest on its own. 

The alternative approach commonly utilized in industry was left out of the scope in research field due to several restrains. Major characteristic of the process was a massive—as compared to the foremost—amount of the energy flow through the sample that is reflected in the widely used name: Hard anodization (HA). A basic constraint that limits access to certain benefits of the process is the amount of heat generated during formation of the alumina, related with the Joule’s effect. Reaching the critical point may result in the electric breakdown that can lead to the destruction of the sample [107]. The discovery of Lee and co-workers renewed the attention to HA [24]. Principle of the discovery was based on the formation of a thin—400 nm—layer of porous alumina prior to the introduction of the high potential. This ‘scaffold’ prevented the breakdown enabling the uniform NAA growth. It was hypothesized that such a pre-patterning promoted the uniform pore nucleation preventing catastrophic events and defects. Growth of the oxide film with this method was also much faster. Recently, even faster growth of NAA film in a process named ultra-hard anodization Noormohammadi et al. [108]. A 58 µm thick membrane was formed in 80 s: 30 times faster than during hard anodization and 450 times faster than with mild anodization. It was possible due to the high current density (2400 mA cm^−2^) combined with control of the barrier layer temperature and the diffusion length to mitigate burning and the dielectric breakdown. 

The new self-ordering regime that exhibits different current/voltage over time characteristics brought questions about differences in the formation mechanism. Detailed analysis of both regimes using voltametric and microscopic methods was provided by Vega et al. [109]. They point out the difference in the local ion concentration as a major factor for the observed distinction in current characteristics. During mild anodization, the ionic concentration remains stable. However, rapid growth of the structure during hard anodization leads to the local depletion that is reflected in the gradually decreasing current when a constant potential is applied—with hard anodization being controlled by the diffusion. Intriguingly, the potential at which the breakdown occurs depends on the initial sample preparation and experimental conditions [24,84,109].

**Figure 6:**
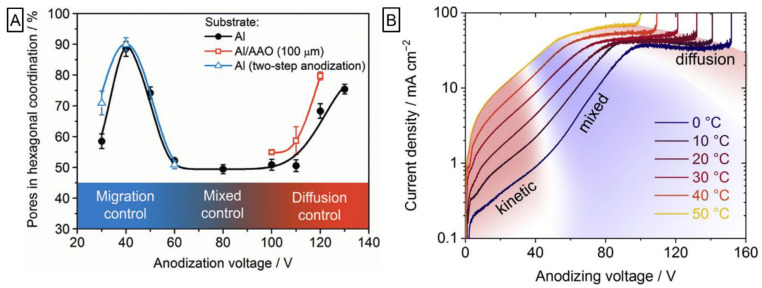
Anodization potential influence on degree of self-ordered growth and voltage/temperature dependent formation kinetics of 0.3 M H_2_C_2_O_4_ anodization of aluminum. (**A**) Dependence of pore ordering degree during formation of alumina structure with highlighted regions of different growth kinetics. Reprinted (adapted) with permission from [111]. Copyright 2017 Elsevier. (**B**) Linear staircase voltammograms with highlighted growth kinetics. Reprinted (adapted) with permission from [113]. Copyright 2019 Elsevier.

The significant contribution to the field was brought by the group of Napolskii and co-workers. They provided analysis that linked the impact of voltage ramp on the morphology and thickness homogeneity. It was demonstrated that when faster 5.0 V s^−1^ ramp was applied instead of conventional 0.5 V s^−1^, the significant reduction of morphological defects is observed [110]. While the occurrence of self-ordering regimes was well-known for spectrum of electrolytes, the question ‘why’ remained unaccounted. An attempt to empirically unravel this behavior was carried out by Roslyakov et al. [111]. They performed the anodization slowly raising the potential (0.5 V s^−1^ for 30–130 V for H_2_C_2_O_4_ and 50 mV s^−1^ for 15–60 V for H_2_SO_4_) and continued at every potential value so 105 C of charge can be utilized (corresponding to ~50 µm thick NAA) followed by the separation of alumina film into thin slices. Upon analysis of the NAA morphology, they proposed a model in which a high level of self-ordering can be achieved in two distinct regimes: The growth rate being restricted by ionic migration through the barrier layer or by diffusion inside the pore. Outside of these frames—during the “mixed” control—pore growth is disordered. Prevalence of the self-ordering depends on the applied voltage and is shown in Figure 6. The applied potential is linked with the formation efficiency, volume expansion, and content of the electrolyte impurities for 20–130 V anodization in 0.3 M H_2_C_2_O_4_ and 19–60 V 0.3 M H_2_SO_4_ [112]_._ The formation efficiency and the volume expansion were found proportional to the potential increase, while the degree of ion embedding was the highest for moderate values exceeding ones achieved with either low or high potential. Recently, detailed analysis of the electrolyte temperature effects on the alumina formation in oxalic acid at mild and hard anodization was provided [113]. Concluding the current profile, higher electrolyte temperature resulted in a shift of both kinetic and diffusion regimes to lower voltages (Figure 6). The temperature increase from 0 °C to 20 °C raised formation speed significantly: 3.8 times for mild anodization and 2.1 times for hard anodization. However, the faster growth was accompanied by the lower formation efficiency and the number of pores grown in hexagonal coordination. The most striking case was that of thicker films (above 20 µm) for 20 °C 40 V anodization as the formation switched into mixed regime explicitly retarding self-ordering. 

### 2.5. Pore Separation Phenomenon

Under normal circumstances, there is no gap between adjacent pores. However, the anodization under high potential may lead to a rarely occurring phenomenon described as a cell separation. Such an incident usually appears during the anodization in sulfuric acid in the narrow range of conditions and materialize with a formation of a less dense matter at the cell boundaries [84,114]. As an outcome, structural integrity of the structure is decreased, reflected in inferior mechanical properties. To date, there is no clear experimentally supported evidence explaining the exact mechanism behind the formation of anodic alumina and this phenomenon. One of the prevalent, widely accepted explanations was the field-assisted dissolution theory first proposed by Wood et al. [27]. However, recent discoveries such as observed morphology of terminated nanotubes [115] and oxide growth locations [116] provided some insight that contradicts the long-accepted theory in some aspects. Moreover, the explanation pointing on the equilibrium between the dissolution and oxide growth as a sole mechanism of the pore initiation does not explain the presence of the anion-contaminated layer at the bottom—as formation of pores should follow the dissolution of the anion-contaminated layer, thus the lack of its presence. It can be further supported with the observation of a double-walled character of the nanoporous anodic alumina structure, where it is the inner wall that contains incorporated anions [32]. In addition, the formation of weaker triple-cell junctions was not sufficiently justified. These doubts followed the proposal of a different mechanism.

**Figure 7:**
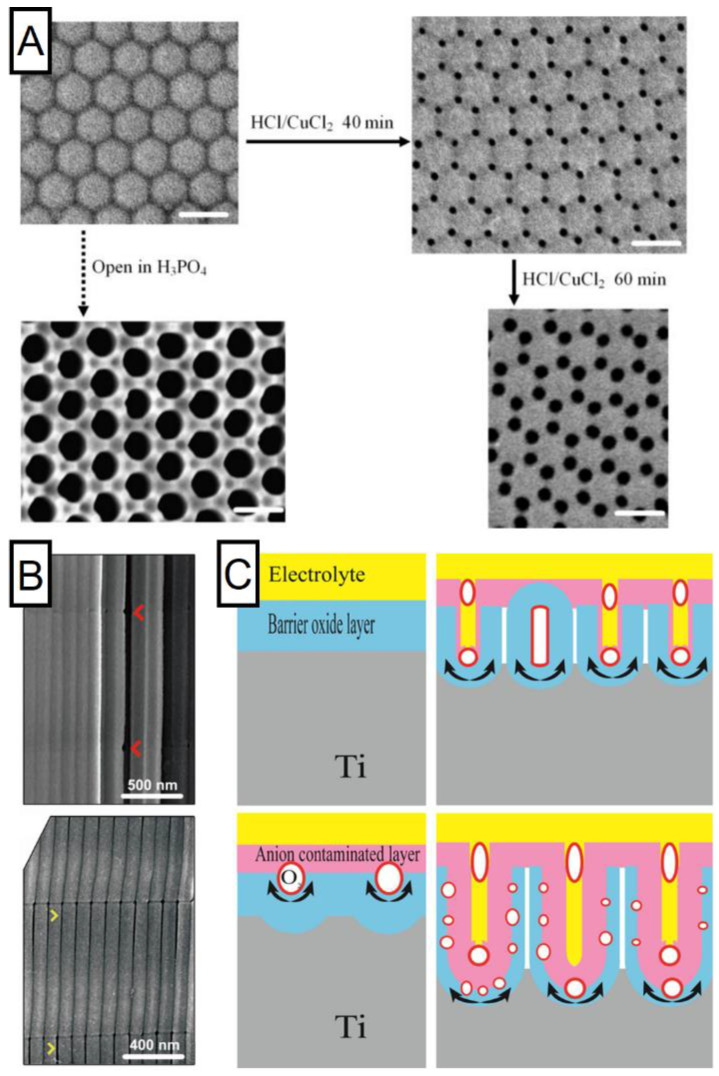
Structure periodical discrepancies and schematic explanation of its formation. (**A**) Selective etching with HCl/CuCl_2_ and H_3_PO4 of structure with local differences in material morphology. Reprinted (adapted) with permission from [118]. Copyright 2007 Wiley. (**B**) SEM images of the structure before (upper panel) and after (bottom) acid etching with visible void formation. Reprinted (adapted) with permission from [122]. Copyright 2015 Elsevier. (**C**) Schematic representation of the cavities formation between double walls of growing titania nanotubes. Reprinted (adapted) with permission from [126]. Copyright 2019 Elsevier.

Importance of the volume expansion during the alumina growth that increases along with electrical field increase was highlighted by Yasumori et al. [84]. This factor combined with lower density of the oxide at the triple cell junction are linked with the lower expansion force in these spots (distribution of the expansion force is circular) [84]. Simulation of the stress distribution along the oxide/metal interface found existence of tensile forces focused near ridges while compressive stress elsewhere [64]. Furthermore, the NAA structure prepared in the conditions promoting cell separation behaves differently upon the chemical etching—such structure shows clear voids around cells that lost its initial hexagonal shape [117]. Interestingly, the mixture of HCl/CuCl_2_ seems to work selectively solely on the three cells junction material, while etching in H_3_PO_4_ provides more uniform etching pattern as shown in Figure 7 [118]. Voids at the cell boundaries seem to be characteristic for the anodization under a high electric field [119]. Additionally, size of the voids has been found to increase with higher voltage [120]. Observation about the distinct chemical composition—hydrated aluminum oxide forms like Al(OH)_3_ and AlOOH—at the cell walls has been supported by Mei et al. [114]. Similar observations that demonstrate ultra-high anodization (620 V) resulting in the formation of unique structure with high fluctuations of outer diameter of the pores were reported in the work of Xinhua et al. [121]. Under certain conditions, formed anodic alumina is not homogenously susceptible to the acid etching, pointing to a different packing density of the structure and/or slightly different composition. Such behavior of the anodic aluminum oxide has been also reported in the work of Wang et al. [122]. As shown in Figure 7, the initially cohesive structure turns into an array of loosely connected pores after a short etching in HCl/CuCl_2_. As mentioned before, a pore separation is usually reported for the anodization in sulfuric acid-based electrolytes. However, it can also occur in different settings. Much more pronounced separation—to the extent of cells being barely connected—has been achieved through anodization in oxalic acid with different addition of ethylene glycol [100,123]. Authors explain the observed pore separation phenomenon by the extensive incorporation of $C_{2}O_{4}^{2-}$ and COO^−^ ions into the NAA structure promoted by the presence of ethylene glycol. Such a structure demonstrates higher susceptibility to acid etching. 

The increased energy flow through the sample results in an intense generation of the oxygen bubbles derived from the water dissociation at the anode interface was proposed by Lee et al. [124]. The importance of bubbles for the formation and shape of the structure formed during the anodization has been reported in the past [117,125]. It can be supported with the observation of closed pores not accessible through the upper surface—the origin of which is not justified by the field-assisted dissolution mechanism—attributed by the authors to the presence of oxygen bubbles. This hypothesis is visualized in Figure 7 [126]. Additionally, oxygen bubbles could force Al^3+^ and O^2−^/OH^−^ ions to migrate around the bubble shaping formation of the NAA. It was proposed that the presence of these species promotes the decomposition of Al(OH)_3_, pointing to the similarity observed during the anodization of titanium [127]. An important discovery that provides a new insight has been brought due to the work reported by group of Zhu [126,128]. For the first time it was possible to observe cavities between double walls of nanotubes, strongly supporting the oxygen bubble formation theory. The cavities distribution contradicts the mechanism of field-dissolution theory and provide new arguments in the discussion regarding mechanism of anodic formation of Ti and Al oxides. Although the adduced discovery has been observed for titania nanotubes, authors connote similarities behind the formation of both anodic oxides [116]. Moreover, the importance of oxygen bubble generation for the formation of the NAA structure has been considered previously already [129]. Certain behavior of the sample cannot be explained by the field-assisted dissolution theory, while supplemented with the recent findings, the oxygen bubble mold theory along with the plastic flow model provide more detailed explanation regarding the formation mechanism.

A better understanding of how nanoporous anodic alumina is formed and improved control over the process lead to many innovations. An important change that has been implemented to tame drawbacks of hard anodization regime was the concept of pulse anodization reported by Lee and co-workers [124]. The intention was to mitigate the heat generation and prevent electrical breakdown enabling to extend process duration—giving another life to the once abandoned strategy. A close observation of the NAA obtained with pulse anodization revealed that nanopore diameter is modulated along the direction of NAA growth precisely following the input voltage. While this work did not report the fabrication of nanotubes, the seminal strategy that originated, coupled with settings promoting pore separation laid foundation for the new material concept—anodic alumina nanotubes (see Section 3.2. Nanotubes). 

### 2.6. Pre- and Post-Anodization Treatments

#### 2.6.1. Pre-Anodization Patterning of the Aluminum Surface

**Figure 8:**
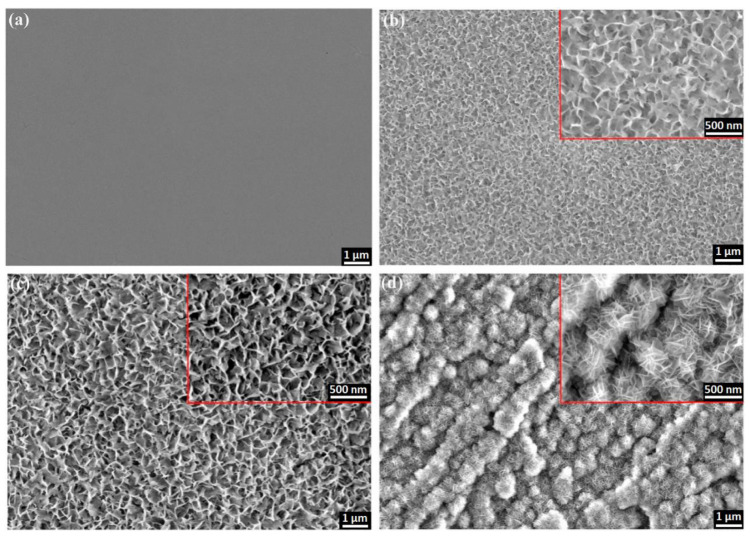
SEM images of hydrous oxide layer formed hydrothermal treatment of aluminum foils at 97 ± 2 °C for (**a**) 0 s, (**b**) 30 s, (**c**) 10 min, and (**d**) 60 min. Reprinted with permission from [130]. Copyright 2020 Elsevier.

The surface of aluminum can be also modified prior to anodization to enhance/guide the formation course. Hydrothermal treatment prior to anodization with intention to produce oxide barrier layer with complex morphology was proposed by Li et al. [130]. Usually, treatment in hot water or water-alcohol solution is applied to seal pores of the already produced alumina and create corrosion-protective layer as the pore entrance collapses. In this case, the idea was to form hydrate aluminum film prior to the anodization. The electropolished aluminum sheet was subjected to hydrothermal treatment in deionized water at 97 ± 2 °C for different time duration. Complex morphology of the obtained hydrate oxide layer was demonstrated to depend on the hydrothermal treatment time as shown in Figure 8. What is more, such a surface was observed to promote the pore nucleation enabling to achieve the fast self-ordering during first anodization step, while the applied potential is outside of a usual self-ordering regime (e.g., 60 V, oxalic acid). This behavior is reflected in the distinct current profile at the beginning of anodization. Additionally, energy consumption during subsequent anodization can be reduced and crystallization of the alumina layer improved.

Apart from common techniques like lithography and selective etching [131], sputtering of aluminum on the surface with desired morphology may be an useful alternative to yield higher structural complexity. The structure made by aluminum sputtering on the 2 µm silica microbeads was reported by Chung et al. [132]. The obtained large-surface area substrate was proven effective for photocatalytic purposes. 

#### 2.6.2. Thermal Annealing

One of the most basic method to affect properties of nanoporous anodic alumina is through thermal annealing. NAA subjected to thermal annealing maintains its mechanical stability and flexibility [133]. As temperature increases first change in the structure can be observed around 700–1200 °C, when it undergoes the rearrangement with gradual formation of γ-Al_2_O_3_. As temperature increases, at 1100–1500 °C α-Al_2_O_3_ progressively emerges. Temperature of the crystalline shift depends also on the electrolyte of choice. For example, the transition of the oxalic-NAA from polycrystalline phase to α-Al_2_O_3_ occurs at 1100 °C, while the sulfuric-NAA needs to be annealed at 1230–1250 °C for such a change to be observed [134,135,136]. Removal of ionic residues depends strongly on the applied electrolyte and tends to be gradual with rapid spikes along the rearrangement of crystalline forms [137,138]. Induced structural changes at the atomic scale are responsible for the increased transparency and hardness, yet mesoporous structure is preserved even above 1050 °C providing for the stability in high-temperature applications [139]. However, while the porous morphology is maintained, deformation of the film can occur (e.g., bending). The approach in which such deformation can be avoided was proposed by Roslyakov et al. [140]. When annealing occurs with the alumina film pressed between two layers of material, the sample maintains its initial shape. When combined with slow temperature increase (~1 °C) around phase transition regions, it is possible to anneal the structure at 1200 °C preserving its mesoporous array forming nanoporous α-Al_2_O_3_ that features superior chemical stability (enhancement by two orders of magnitude). 

## 3. Engineered NAA Structures

### 3.1. Structures Based on the Modulation of the Anodization Current

Since a direct relationship between the anodization voltage and the diameter of NAA is a well-known fact [27,141], possibility of designing its geometry in vertical axis through dynamic, pulse-like alterations of the anodization current is expected. However, the existence of a barrier layer—a layer of the oxide that lays between a surface of the anode and the porous oxide—restricts the way in which diameter of pores can be affected during the process. The barrier layer acts as an insulator that limits the transfer of ionic species and features significant electric resistance retarding speed and range of accessible alterations in the structure through potential control [142,143]. The development and understanding of the pulse anodization mechanism pioneered by Lee et al. brought new fabrication tools to the field. An important improvement came along with the concept of applying high current pulses interlaced with low current density periods [124,144,145,146]. A significant aspect involved a better understanding of porosity levels as well [24]. A variant of pulse anodization yielding periodically Y branched pores is reported by Peng et al. [147]. It was possible due to a saw-tooth anodization current profile and a high current density (above 70 mA cm^−2^). Additionally, structural features could be further altered with the duration of pulses. Applying high density current pulses to tailor optically sensitive structures bear some limitations, though. For example, the growth speed—that can be even a tenfold of that during mild anodization—is not constant in potentiostatic mode due to ionic species deficiency, contrary to mild anodization. What is more, such a process is more prone to induce cracks in the structure during growth, which is the another factor limiting their application for the optical responsivity. The major disadvantage of the mild anodization is definitely its low growth rate (3–8 µm h^−1^), which translates into several days long the fabrication of thick films. On the other hand, the process is much more predictable and enables more precise control during alumina growth. An advantage of NAA fabrication stems from the possibility to tailor-engineer its periodicity in the three dimensions. 

Sinusoidal current alterations can be utilized to fabricate photonic crystals with multispectral photonic stopbands (PSB), that in turn can be precisely tuned [148]. Infiltration of D-glucose into the pores results in a spectral shift of PSBs and the possibility of a quantitative detection based on the shift. In other work, Gaussian pulse anodization with different time gaps between the pulses has been examined [149]. The PSB optical features of the structure such as the position of the central wavelength, the width at half-maximum, and the intensity could be adjusted by altering the time gaps—with the incidence angle of photons and the porosity of the structure being relevant as well. While the previous works involved 0.3 M oxalic acid, investigation regarding properties of photonic structures made with sulfuric acid and various additives (alcohols and polyols) is provided by Lim et al. [150]. The comprehensive study revealed advantages of such approach. Potential benefits of the additives are related to the suppression of dissolution rate during the anodization and the incorporation of impurities containing carbon, both having a significant and positive effect on the quality of forbidden light propagation within the structures. Forty percent addition of methanol provided the best results, yielding the photonic structure with the highest quality factors amongst the compared. 

### 3.2. Nanotubes

The discovery of pulse anodization was a significant innovation providing for the further development of anodic alumina nanofabrication. In-depth analysis enabled to properly utilize hard anodization with its impressive growth rate and a geometry not previously accessible [151]. On the other hand, alteration of the pore diameter in length was a separate concept with its own advantages [124]. For the first time, the fabrication of nanotubes through the hard anodization with pulses was reported in 2008 [144]. The approach consisted of immersing electropolished aluminum into 0.3 M H_2_SO_4_ and the preparation of an initial, thin NAA layer through mild anodization at 50 V. The formation of a thin layer under low potential is important for the prevention of the electric breakdown, which occurs frequently upon immediate surge of the potential when anodizing pure aluminum. It was followed by galvanostatic pulses of 3.16 mA cm^−2^ and 368 mA cm^−2^ interchangeably, where high current pulses provided growth of the structure (tubes), while mild anodization pulses—apart from dissipation of heat—aimed at the production of weaker spots in the structure. These weaker spots are crucial for the selective separation in further parts of the process. The galvanostatic mode has been chosen due to the characteristics of the hard anodization with the gradual current decrease—affecting the growth rate—when constant voltage mode is applied. Such a setup enable the precise tailoring of the nanotubes length. After removal of aluminum, the structure was subjected to the chemical etching in a similar manner to the previously reported selective etching approach [118]. Then, the sample was sonicated to yield a colloid of liberated nanotubes. It is important to note that sonication itself was reported insufficient to provide the efficient liberation of nanotubes. Prior to the discovery of Woo Lee, the fabrication of anodic alumina nanotubes was more complicated and involved techniques like wet-chemical etching [152], anodization of thin layer of aluminum on silicon sheet (Al/Si) [119], hydrothermal synthesis with nanowires of a different material as a sacrificial template [153,154], atomic layer deposition [155,156], and a few more [157,158]. Moreover, these methods carried many disadvantages. Apart from a more complex protocol, reproducibility and ability to precisely tailor shape of the nanotubes was strongly limited. Nanotubes made of alumina display plenty of attractive features making them valuable candidates in fields like nanoelectronics, catalysis, or biomaterials. After fabrication, they exhibit a low surface reactivity and lack catalyst contamination. Their surface rich in hydroxyl groups facilitates functionalization through salinization. Additionally, the initial closed nature of the structure enables selective modification of outer/inner interface without complex procedures. In neutral pH DI water, nanotubes are positively charged, while in PBS, they exhibit the negative charge. The absolute charge value is moderate providing for the decent stability and possibility of electrostatic interactions with proteins and other nanoparticles. What is more, singular elongated cell of the structure fabricated in similar conditions display significant plasticity pointing on certain level of material’s flexibility [159].

Several years from the discovery carried out by Lee et al. [144], the topic of anodic alumina nanotubes has been brought back and significantly developed. The first report was a systematic study with an aim to evaluate an in vitro nanotoxicity of AANTs by Wang et al. [160]. Nanotubes of different length—ranging between 0.7 and 5.8 µm—were tested with RAW 264.7 mouse macrophage cells and MDA-MB 231-TXSA human breast cancer cells. The reported toxic window of 7.8 for AANTs was shorter than that of many other high aspect ratio materials. It was followed by detailed investigation of the fabrication process with the scope on the electrochemical aspect of the nanotubes [122]. The focus of the work was to understand the formation mechanism to optimize the process and improve its final efficiency. The fabrication protocol has been expanded with the formation of the protective/functional NAA layer—that facilitates the sample handling, and the improvement of the nanotubes separation. What is more, 10% addition of ethanol to the electrolyte during pulse anodization has been proposed to promote the Joule’s heat generation associated with the increased yield of the process, obtaining even shorter nanotubes. In the next report, performance of AANTs as a 1D drug carrier has been examined [161]. The Apo2L/TRAIL model drug has been successfully loaded inside nanotubes and delivered into cancer cells inducing apoptosis. An exceptional loading capacity for the Apo2L/TRAIL protein was estimated on 104 ± 14.4 µg mg^−1^ of AANTs supporting its promising application as a nanocarrier. The gathered findings enabled to propose AANTs in a proof-of-concept cancer therapy based on the signaling network targeting [162]. The aim was on the autophagy and endoplasmic reticulum paths of the primary human foreskin microblasts and the human monocyclic cells. AANTs showed a promising utility in the role of a non-toxic non-degradable nanomaterial. What is more, it was possible to follow the intake mechanism of the material. The last work involved topic of in vivo toxicological and pathological studies in eight-week BALB/c immune competent mice. AANTs were introduced through an intravenous injection (IV) and a subcutaneous implantation [163]. The IV approach did not show any impact on body weight and mortality of mice during 28 days at doses between 20 and 100 mg kg^−1^, and nanotubes were found to accumulate in liver and spleen. The highest dose, however, induced moderate hepatotoxicity. The subcutaneous introduction—on the other hand—led to an inflammatory response. It is important to note that a surface coating—for example with PEG—should improve biocompatibility of the material indicating need for further experiments. 

**Figure 9:**
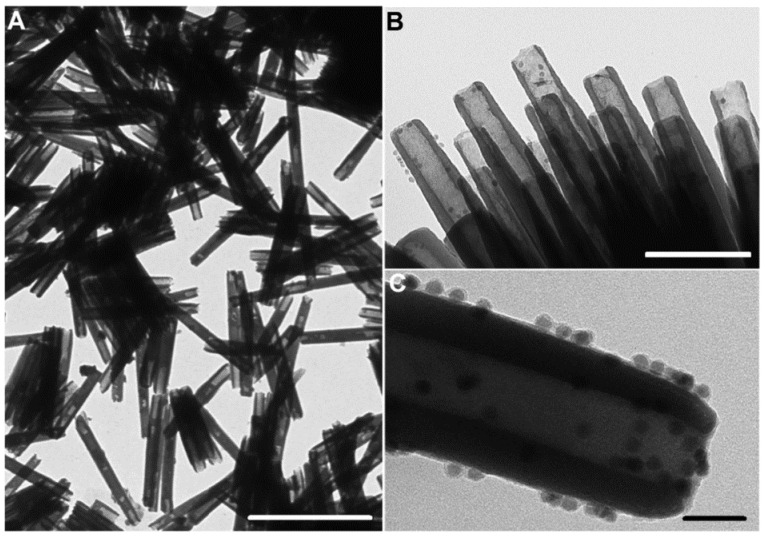
TEM images of anodic alumina nanotubes (AANTs). (**A**) Unmodified AANTs, (**B**) maghemite decorated AANTs (MAANTs), and (**C**) magnified capture of MAANTs; scale bars: 1 µm, 200 nm, and 50 nm respectively. Reprinted with permission from [165]. Copyright 2020 Elsevier.

A step forward in the fabrication of AANTs defining in detail the relation between current/voltage input and geometrical features of nanotubes was reported by Domagalski et al. [164]. Apart from defining the relation between pulse duration and the resulting nanotube length, they discovered that current density during hard anodization pulse can affect the outer diameter and the surface charge of AANTs, contributing a higher control of the process and enabling tailor-engineering of anodic alumina nanotubes. Moreover, optimization of the technology allowed to yield even shorted nanotubes than before—424 nm in length on average. Their next work [165] involved decoration of nanotubes with maghemite superparamagnetic nanoparticles to yield magnetic nanotubes (MAANTs) combined with functionalization inside the nanotubes. The structural integrity preceding the separation was utilized to conduct the selective modification of inner walls with the padding of a protein functionalized with the fluorophore. Later, nanotubes were connected to magnetic nanoparticles through electrostatic. TEM images of native nanotubes and produced composite are shown in Figure 9. The detection system was aimed at the recognition of enzyme molecules, demonstrated with cathepsin B and based on the enzymatic cleavage of the fluorophore-modified protein and the release to the medium. Magnetic maneuverability was used to separate nanoparticles from the main volume of the medium. 

### 3.3. Micro- and Nanoparticles

**Figure 10:**
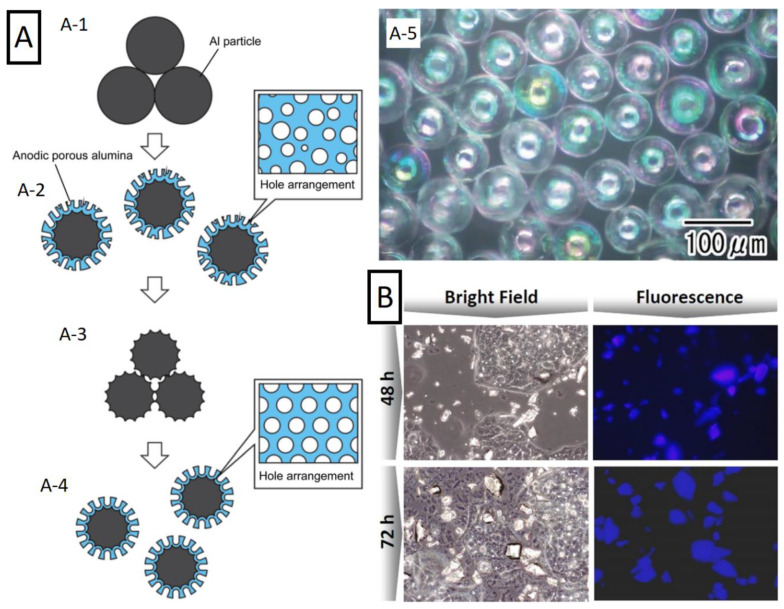
Nanoparticles based on NAA. (**A**) (**A-1**–**A-4**) Schematic of the multi-step formation process of nanoporous anodic spheres and (**A-5**) optical microscope image of hollow spheres made of porous alumina obtained by removal of aluminum residues. Reprinted (adapted) with permission from [173]. Copyright 2018 The Royal Society of Chemistry. (**B**) Microscope images of HepG2 cells incubated with intrinsically photoluminescent nanoporous alumina particles obtained through grinding of NAA substrate. Reprinted (adapted) with permission from [166]. Copyright 2015 American Chemical Society.

A prevalent perception of nanoporous anodic alumina is as a 3D nanostructured material and utilized as such—with vast possibilities described before. In the field of nanofabrication, NAA is mostly used as a template due to its high surface area and periodic regularity. However, anodic alumina itself exhibits a plenty of promising properties that could make derived nanoparticles attractive in ways not accessible for the plain NAA. Apart from the nanotubes produced with the pulse anodization (Section 3.2), there are other alternatives. An approach consisting of mechanical grinding of the alumina membrane, obtaining nano/micro-particles with the structural features of nanoporous alumina was reported by Xifre-Perez et al. [166]. These particles exhibited luminescence and were surface functionalized with antibodies. Their performance at different concentrations was demonstrated with HepG2 cells showing good biocompatibility. Microscope images of the cells incubated with alumina micro particles are shown in Figure 10. Further studies provided more comprehensive analysis of alumina micro- and nanoparticles obtained with different methods [167]. Similar strategy to obtain alumina microparticles was employed in the work of Chen et al. [168]. However, in this case, the base material was NAA-based Bragg reflector. Particles were prepared by a mortar-grinding of the alumina film and the subsequent sonication. Obtained microparticles retained their optical properties—the same as the initial template—a promising prospect with regards to microsensors. 

Another example of smart approach is the fabrication method developed by Matsuda et al. The major thought behind this idea is the preparation of aluminum in the structural shape similar to the desired outcome. One example can be alumina nanowires. At first, nanoporous alumina molds with various geometrical features (pore diameters of 15–340 nm and cell size between 30 and 500 nm) were used to form nanowires on the surface of aluminum by a applying pressure of 7 GPa, followed by the acid-dissolution of the mold to liberate the nanowires. These structures were later anodized in a similar way to the standard NAA (0.3 M H_2_SO_4_ at 0 °C under 12 V for 2–10 min). As result, nanowire arrays made of nanoporous anodic alumina with and without Al core were yielded [169]. A similar strategy was also conducted using aluminum microspheres. Al particles were introduced to a cylindrical holder with a porous membrane at the bottom [170]. Then, Al electrode was inserted applying appropriate pressure to ensure electrical connection between the particles. Anodization conditions (1 M HCl + 3 M H_2_SO_4_) were aimed at etching of the aluminum surface with deep pits rather than formation of regular nanoporous structure. At this stage, aluminum layer was selectively removed to yield etched aluminum microspheres. It is worth noting that the contact point between the spheres remained unchanged as the electrolyte could not penetrate this part. Furthermore, authors also performed standard mild anodization on these etched aluminum spheres followed by dissolution of aluminum obtaining nanoporous oxide replica of the spheres. Their next work continued this idea but introduced a two-step anodization in order to provide ordered nanoporous alumina layer on the surface of aluminum spheres (illustration of fabrication process is shown on Figure 10) [171]. Various anodization conditions were utilized in order to yield different NAA geometry. After the etching of the aluminum core, hollow NAA spheres were obtained—presented in Figure 10. The pits that can be observed on the surface of these structures are remnants of spots that were not anodized due to continued contact between aluminum spheres. Moreover, these spheres were later modified through electrolytic deposition of Au, forming a composite. These nanoparticles are an alternative in the field heavily dominated by mesoporous silica [172].

Decades of experiments aiming to understand the NAA formation mechanism enabled to turn the surface functionalization approach into a precise nanofabrication method. The versatility is also reflected in a broad range of reported applications that were based on nanoporous anodic alumina. 

### 3.4. Funnels and Inverse Funnels

**Figure 11:**
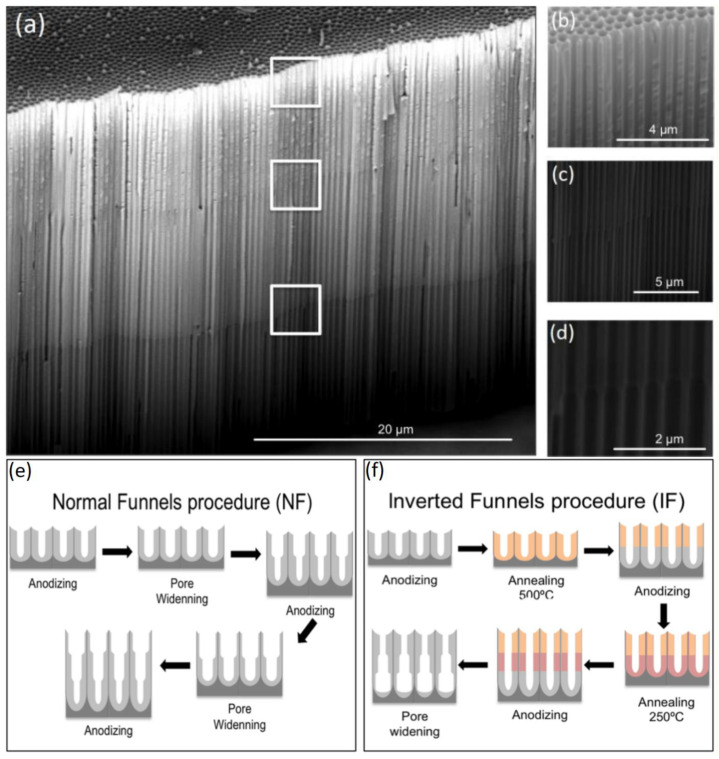
Cross-section ESEM images of inverted funnel NAA structure and fabrication schematics. (**a**) Entire structure, (**b**–**d**) magnified images of transitional regions, (**e**) fabrication procedure of funnel morphology, and (**f**) inverted funnels morphology. Reprinted (adapted) with permission from [175]. Copyright 2017 MDPI.

This alternative involves smart combination of anodization, annealing, and chemical etching allowing the production of exceptional features. Two distinct ways in which such a structure can be obtained were demonstrated by Santos et al. In the first approach, a diameter gradient was obtained applying several anodization steps [173]. After every step, the sample was subjected to the pore widening through chemical etching with 5% H_3_PO_4_. That way, it was possible to obtain the structure with a gradual decrease of the pore diameter until the pore bottom. During the second experiment, it was possible to form a reversed gradient with the diameter increase towards the pore bottom [174]. It was achieved through annealing of the sample after every anodization step with the highest annealing temperature applied to the first, external layer and applying lower annealing temperatures with each consecutive step. Then, the sample was subjected to the 5% H_3_PO_4_ chemical etching. The optical properties of NAA enabled to observe the formation of nanofunnels in situ in real-time by means of RIfS. Similar structures were later examined by Porta-i-Batalla et al. [175] to determine the influence of the pore geometry on the drug load and its release performance. Figure 11 shows the morphology of reversed funnel architecture captured with ESEM (environmental scanning electron microscope) and the fabrication schematic of both normal and inverted funnels. Another example in which anodization steps are interlaced with annealing to obtain bottle shaped pore tips has been reported by Liao [176]. After two standard anodization steps, samples were annealed at 500–600 °C, reanodized, and chemically etched in phosphoric acid (with potentiostatic in situ monitoring). As result, it was possible to form NAA regions with two highly different pore diameters: 220 and 345 nm. 

With a different approach to alter the pore diameter, an improvement of the process control by mitigating the tendency to form branched pores that are commonly formed upon exponential decrease of anodization potential was demonstrated [177]. The development of the process was a consequence of attempts to achieve a thinner barrier layer and allowed to tailor a step-less architecture with a gradually decreasing pore diameter. This was possible due to the combination of high electrolyte temperature and fast voltage drop—brief enough not to interrupt the anodization process. The combination of several processing procedures is important to exceed the limitation of a standard two-step anodization and yield more complex architectures. 

### 3.5. Hierarchical Pore Structures

The structure resembling funnel-like characteristics but also some hierarchical features was reported in the work of Liu and Biring [178]. The aluminum surface was pre-patterned with the nanotube array interlacing shallow and deep patterning. After the first anodization step, the second one is performed with higher anodization potential—twice the initial level—forcing interpore distance to increase twice and terminating the growth of many nanochannels. The resulting structure consists of long-funnel like pores surrounded by short, narrow pores that ends just as the former expands. 

**Figure 12:**
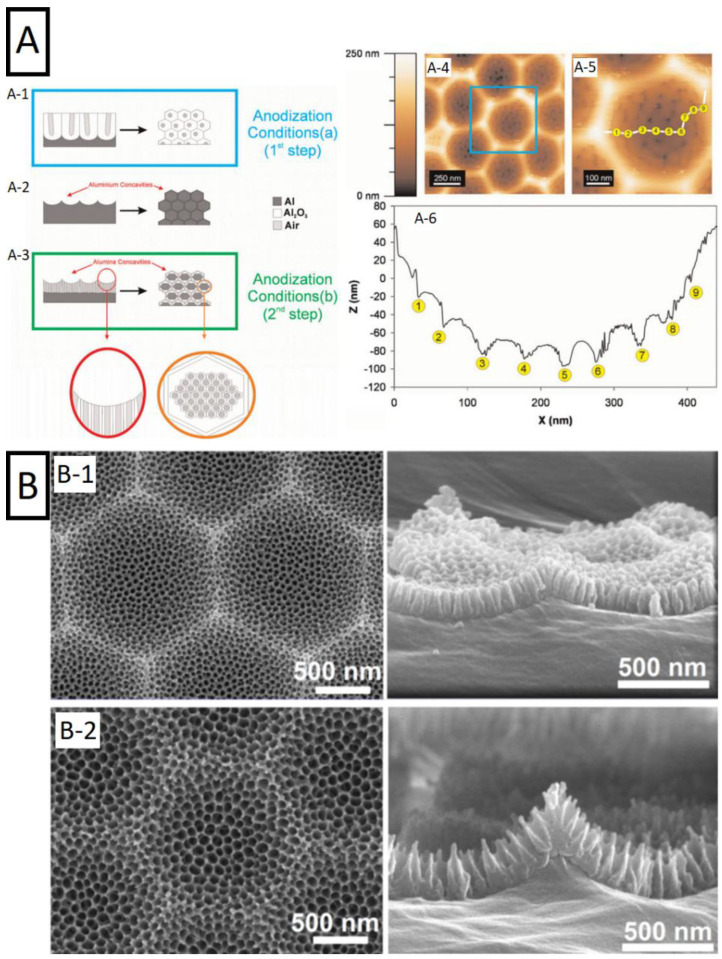
A morphology of hierarchical pore structures. (**A**) (**A-1**–**A-3**) Fabrication steps of hierarchical nanopore array, (**A-4**,**A-5**) AFM (atomic force microscope) top side views of hierarchical nanoporous anodic alumina, and (**A-6**) cross-section of the corresponding image. Reprinted (adapted) with permission from [29]. Copyright 2010 Wiley. (**B**) SEM images of tapered nanopores obtained by mild anodization in different electrolytes. (**B-1**) 0.3 M sulfuric acid, 20 s (d_int_ = 50 nm) and (**B-2**) 0.3 M oxalic acid, 40 s (d_int_ = 95 nm). Reprinted (adapted) with permission from [180]. Copyright 2019 Elsevier.

A more pronounced hierarchical morphology can be achieved with a different approach. Usually aluminum anodization is carried out by repeating similar conditions twice—concavities left after removal of the oxide film resemble geometrical features of the oxide grown during the second step, ensuring its high regularity from the beginning. However, growing behavior is different when conditions of the second step differ. Such strategy—aimed at smaller pores grown during the second step—is presented in the work of Santos et al. and illustrated in Figure 12 [29]. The growth of the pores is organized inside previously produced concavities. Different anodization conditions such as voltage, electrolyte, and the process temperature were examined yielding various geometries. Morphology of the structure is shown in Figure 12. Such an approach may serve as an alternative to less accessible lithography pre-patterning, when more complex surface morphology is needed. Size of concavities did not exceed 1 µm in the report [29].

Such possibility was reported later, with the work of Jin et al. [179]. Not only the size of the initial patterning on the aluminum surface increased, but also more complex pore profile was achieved. Focusing on the anodization voltage changes, the combination of previously presented idea with periodic pulse-like anodization was utilized. As result, the structure features hierarchical morphology with pores ordered along the cavity, while the profile of the pore became serrated. The idea is further continued with the introduction of more anodization steps by Ma et al. [180]. The surface has been provided with a bowl-like array through the stable anodization in citric acid, followed by the combination of multiple mild anodization steps and the chemical etching. Architecture of such tapered pores obtained with oxalic and sulfuric acid is shown in Figure 12. Apart from a more complex morphology, tendency of such pores to grow aligned with the radius of each cavity—perpendicularly to the outer surface—was observed. It was suggested that initial oxide film is thinner under low anodization voltage, which—due to less localized concentration of the electric field—results in nanopore nucleation, more homogeneously on the whole surface of the curvature rather than on the bottom of the curvature. Furthermore, authors compared pore density when grown on flat and bowl-like surface. Curvature of the surface enabled to yield 1.5 times higher packing density (1.68 vs. 1.15 × 10^15^ cm^−1^).

### 3.6. Three-Dimensional Interconnected Nanoarchitectures

**Figure 13:**
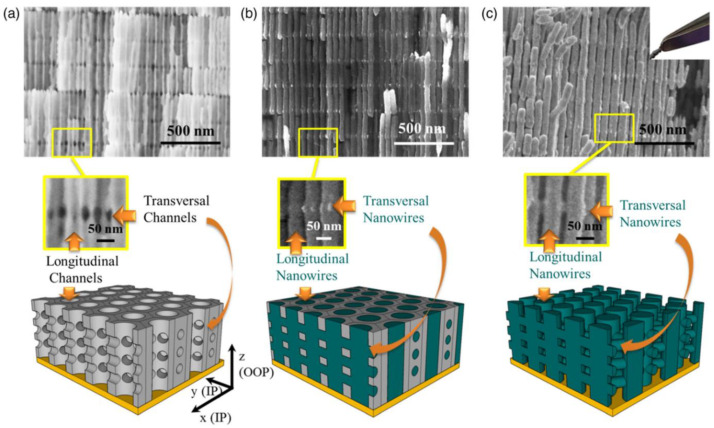
SEM images and schematics presenting template-assisted fabrication of 3D magnetic alloy network. (**a**) Raw alumina template, (**b**) structure infiltrated with magnetic alloy, and (**c**) 3D network of magnetic nanowires. Reprinted with permission from [183]. Copyright 2019 Wiley.

A different approach to pulse anodization is presented in the work of Martin et al., in which they report the fabrication of 3D nanotubular network [181]. Contrary to the previously mentioned examples, such structures feature not only periodic modulation of the pores, but also the connection between the pores through transversal nanochannels. Geometrical features are controllable through the anodization, accessible film thickness reach dozens of microns and the array is highly regular. Process conditions were inspired by seminal discoveries of Lee et al. [124,146], introducing some differences. Pulses of potentiostatic mild anodization were interlaced with galvanostatic hard anodization at moderate current densities. An important principle during the design of the process was to preserve the longitudinal continuity of the pore arrangement. For that reason, hard anodization pulses were adjusted to yield structure with the same interpore distance. Initial attempts involved potentiostatic hard anodization pulses at 36 V—that quickly resulted in severe damage to the structure as the process continued. The long-range regularity of the structure was possible when moderate galvanostatic hard anodization pulses were applied (27.5 mA cm^−2^). After anodization, chemical etching in phosphoric acid was performed, partially removing the oxide layer grown during hard anodization pulses and forming transversal nanochannels [181]. Different etching rates for the structure parts formed in different anodization regimes allow the distance between consecutive interconnected planes to be tailor-engineered by adjusting the pulse duration. This idea was later continued with series of works throughout the years. Thermal conductivity of these alumina networks and the possibility to tailor the conductivity through changes to the structure geometry and filler materials was reported by Abad et al. [182]. Properties were demonstrated to depend on the thickness of the membrane, the number of nanochannels, and the interchannel distance of the membrane. Metallic fillers provided the electrical conductivity in the vertical plane with the decrease of thermal conductivity as compared to the filler material itself. Later, a structure made with Co_x_Ni_1-x_ alloys using NAA with transversal nanochannels as a sacrificial template was reported by Ruiz-Clavijo et al. [183]. After anodization and selective etching, 3D-NAA templates were coated with a layer of chromium and gold through evaporation. These coated templates were used as working electrodes for electrochemical deposition, when the alloy was deposited. Then, the alumina template is removed revealing the 3D network of magnetic nanowires. Steps of the fabrication process and changing structure morphology is shown in Figure 13. The report also describes how magnetic anisotropy of the network can be modified with changes to the structure geometry.

Valuable optical properties of these structure are the result of the geometry. These unique alumina networks features intrinsic reflections bands, that can be tailor-engineered in full range of visible light [184]. What is more, the structure features sensitivity to environmental changes such as material wetting. In a different example, process conditions are used to fabricate Bragg reflectors and alumina used as a sacrificial template [185]. The structure is infiltrated with polyethylene under low vacuum following by the removal of aluminum and alumina template. As a result, flexible Bragg reflectors made of nanostructured polymer are obtained. 

## 4. Examples of NAA Applications

There are many fields in which nanoporous anodic alumina structures demonstrated their utility. To provide overview of many functionalities discussed in Section 4.1, Section 4.2, Section 4.3, Section 4.4, Section 4.5, Section 4.6 and Section 4.7 they are gathered in the Table 2. 

### 4.1. Photonic Structures

Optical properties of NAA are amongst the most impressive. Glass-like transparency, chemical resistance, and complex, tailorable nanomorphology makes of NAA a valuable material for optical applications. In fact, this is one of the most investigated use of nanoporous anodic alumina. Nanoporous anodic alumina can be also designed as a photonic crystal (PC)—usually through sinusoidal anodization. A detailed description of this role has been already provided in several reviews [34,46,186]. 

PCs are structures featuring a periodic variation of their refractive index, interlacing regions with high and low refractivity. As an effect, some wavelengths can propagate in the structure while others cannot—these are described as the photonic band gaps. It is due to multiple scattered interferences—defined by Bloch modes—that alter the flow of electromagnetic waves [187,188,189]. Propagation of light can be adjusted through a change in the geometry. The most common PC structures made this way are Fabry-Perót interferometers [190], gradient-index filters [191], distributed Bragg reflectors [192], and optical bandpass filters [193]. Their major utilization is as optical sensors. An exemplary application of such sensor is a real-time monitoring of the interaction between human proteins and heavy ions [194]. 

**Figure 14:**
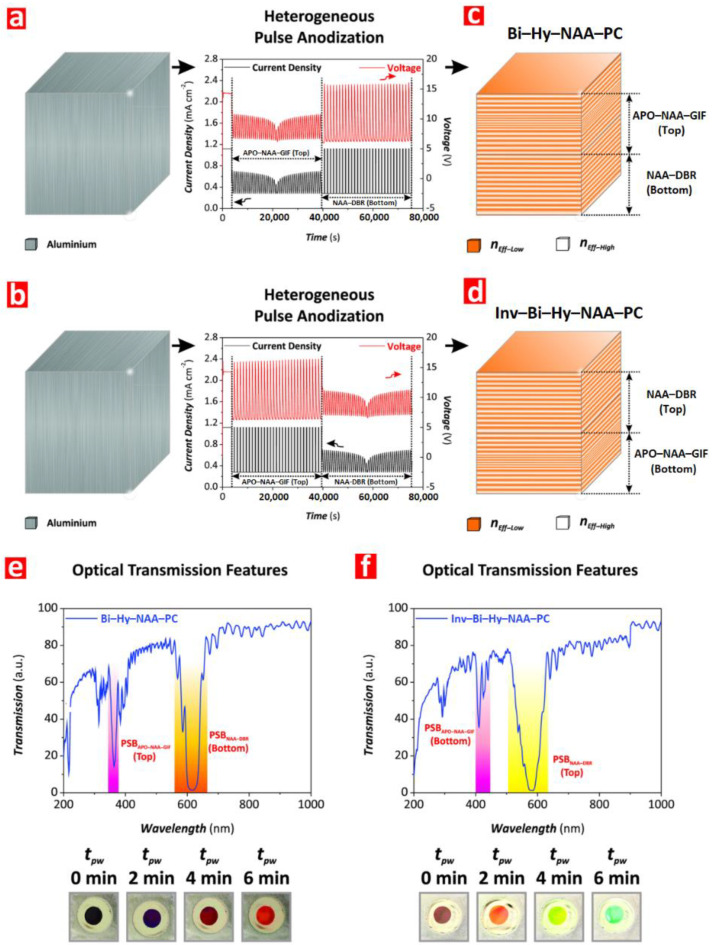
Fabrication and optical properties of hybrid photonic structures. (**a**,**b**) Anodization profiles, (**c**,**d**) resulting structures composed of two and three distinct photonic layers, and (**e**,**f**) measured transmission spectra with highlighted photonic stopbands and digital pictures demonstrating interferometric color of the PC structures depending on the pore widening time. Reprinted with permission from [196]. Copyrights 2019 Springer Nature.

The precise control of the optical properties across the visible—NIR spectrum with pulse-anodized oxalic-fabricated NAA was demonstrated by Acosta et al. [149]. In addition, there are other types of structures as well. Fabrication of structures with a phase shift defect inside the sinusoidal profile of the effective refractive index was reported by Kushnir et al. [195]. Position of the resonance transmission peak could vary between 250 and 1500 nm. Recently, method combining different anodization profiles was reported by Lim et al. [196]. As result, hybrid photonic structures composed of distributed DBRs and apodized GIFs were obtained facilitating for the design of specific photonic stopbands. The anodization profile, morphology of the structure, and resulting optical properties are shown in Figure 14. 

The structure can be provided with an additional functionality when infiltrated with material that serves as a medium different than air, for example by filling the pores with liquid crystals. Thermotropic ferroelectric liquid crystals infiltrated into 10, 15, and 20 nm NAA pores surface-modified with a polymer (the native oxalic NAA surface does not provide sufficient stability) were reported by Busch et al. [197]. The pore diameter of obtained nanohybrid materials was found to be linearly proportional with the electro-optical birefringence which affected the relaxation frequency. Group efforts done by Huber and Schönals teams with co-workers investigate the effects of liquid crystals confined in nanoporous structures—nanoporous anodic alumina and mesoporous silica. Several works of Yildirim et al. investigated a behavior of discotic ionic crystals in confined porous space. Measurements included analysis of a molecular mobility (broadband dielectric spectroscopy and specific heat spectroscopy) [198], a phase behavior, and a molecular ordering (differential scanning calorimetry and dielectric spectroscopy) [199]. 

**Figure 15:**
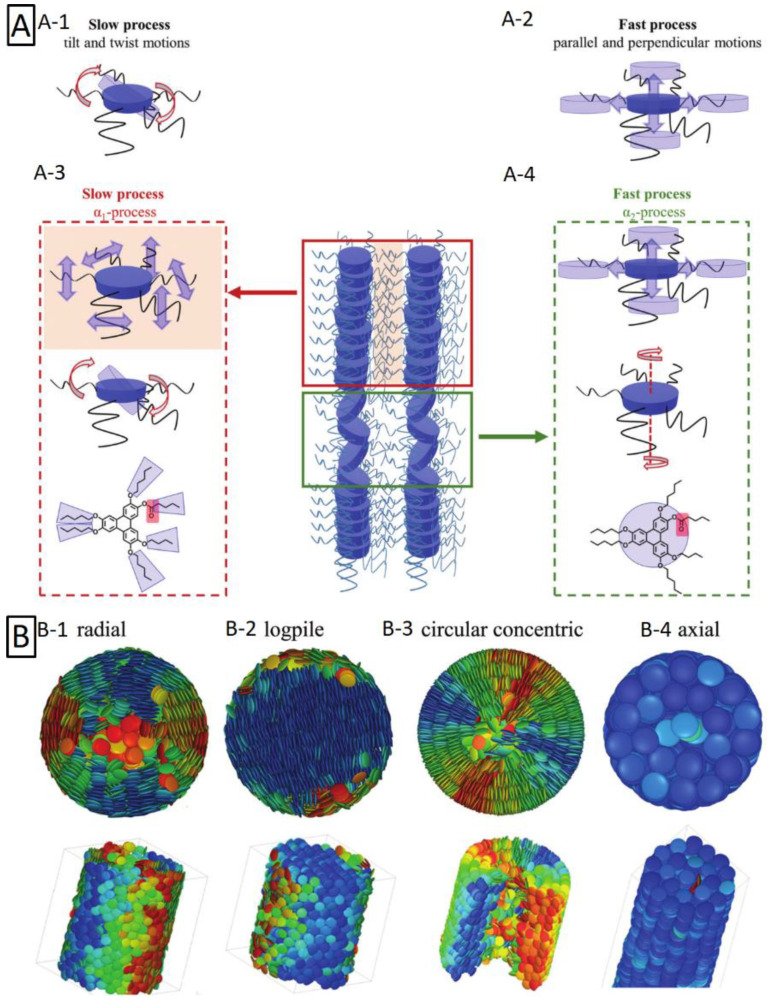
Motion and possible distribution of discotic liquid crystals in nanopore-limited space. (**A**) Illustration of (**A-1**) tilt and twist and (**A-2**) parallel and perpendicular motion of single discotic molecule. Cooperative molecular fluctuations and related changes to the column packing are assigned to (**A-3**) α_1_ and (**A-4**) α_2_ processes. Blue highlight on the structural formula represent parts assigned to glassy dynamics while red coloration represent parts of the molecule that enable to sense this glassy dynamic through Broadband Dielectric Spectroscopy. Reprinted (adapted) with permission from [200]. Copyright 2019 The Royal Society of Chemistry. (**B**) Monte Carlo simulation showing snapshots of columnar discotic order in cylindrical pores (**B-1**–**B-4**) in top- and side-view for different arrangement of discotic particles. Reprinted (adapted) with permission from [201]. Copyright 2019 The Royal Society of Chemistry.

In recent experiments, the focus was directed on understanding a charge transport in such systems [200]. Discotic liquid crystals were investigated with regards to molecular dynamics and phase behavior. Multiple glassy dynamics were detected thanks to detailed analysis. It was possible due to dipole functionalization. Figure 15 shows different types of discotic crystals motion than can occur in highly confined spaces. This finding can constitute for a next step in the future of molecular electronics. Further, self-assembly of discotic liquid crystals in NAA was presented in the work of Sentker et al. [201]. Nanoporous structures have features significantly smaller than visible light wavelengths. This provides for their potential use as a photonic metamaterial—a structure in which optical functionality is determined by its geometry rather than by the material composition itself. Introduction of liquid crystals to these confined spaces can expand possibilities of such structures to interact with light. Liquid crystals exhibit optical birefringence affected by their organization, that can be adjusted through the chemical character of the structure, its pore size and temperature. Optical properties of the porous material with liquid crystals with regards to pore diameter, pore surface functionalization, and temperature were evaluated. Modification of the optical properties through these conditions was also demonstrated. Monte-Carlo simulation of columnar discotic order in confined space of nanopores is shown in Figure 15.

### 4.2. Sensors

**Figure 16:**
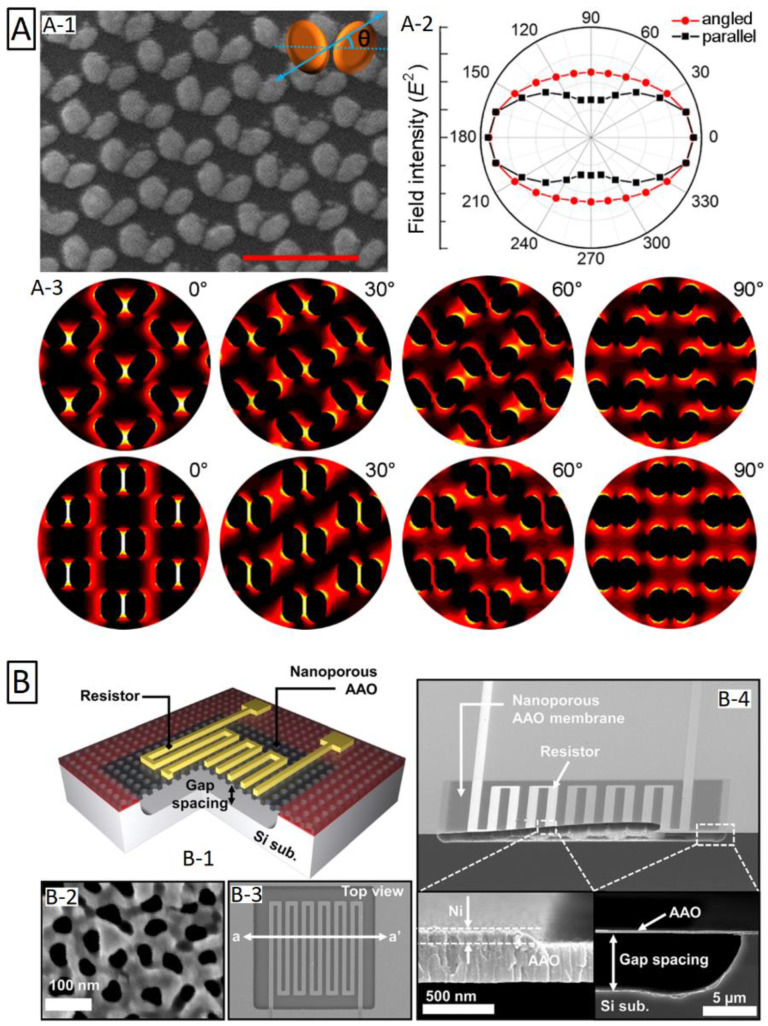
(**A**) Preparation of oriented nanoparticle dimers using NAA as a shadow mask. (**A-1**) SEM image of ellipsoidal dimers angled at 60°; (**A-2**) calculated electric field density with regards to dimers’ angular orientation; and (**A-3**) FDTD simulation of electric field distribution of dimers at different conformation. Reprinted (adapted) with permission from [204]. Copyright 2017 American Chemical Society. (**B**) (**B-1**) Schematic of Pirani sensor and SEM images of (**B-2**) NAA membrane top view, (**B-3**) Pirani sensor top view, and (**B-4**) cross-section captures of fabricated device. Reprinted (adapted) with permission from [214]. Copyright 2016 AIP Publishing.

In recent years, the growth of publications reporting the use of NAA as a photonic structure can be observed. The value of its unique geometry has been demonstrated in many fields, for example as sensing platforms [202,203] providing a promising alternative over plasmonic nanoparticles. The idea to utilize NAA as a shadow mask to fabricate a surface covered with plasmonic dimers was presented by Schmidt et al. [204]. At first, thin (~250 nm) alumina film was subjected to removal of the barrier layer and transferred onto the surface. Different pore widening time was applied to yield various pore diameters. The substrates were placed on titled substrates and shadow deposition was performed at different angles. Through configuration of fabrication process, it was possible to control deposition for each particle of dimer pair independently providing promising ability for tailoring plasmonic surfaces. ESEM showing exemplary dimer, variation of the calculated electric field density in relation to the laser polarization angle, and FDTD simulation of electric field distribution for angled and parallel dimers under different excitation polarization are shown in Figure 16. Interactions with light are also utilized for practical application of NAA interferometers with purpose of copper detection [207]. In a different approach, graphene layer deposited onto the NAA surface through chemical vapor deposition serves as a platform to deposit an analyte [205]. When silver nanoparticles were introduced to the system, the platform combined both surface enhanced Raman scattering and interference amplification. 

The surface of NAA can be also easily modified with several functionalization protocols such as silanization [206]. What is more, NAA’s enormous surface area facilitates an efficient utilization of the material, which can be especially attractive in more sophisticated or real-time detection. Geometrical features of the NAA structure grafted with the streptavidin-biotin complex were examined by Pol et al. [208]. The setup was implemented into a flow cell system to provide real-time monitoring. In addition, an aptamer padding proved to be a valuable solution [209]. Works of Tabrizi et al. show vast versatility of such structures that are capable of the precise recognition of specific biomolecules and ions: determination of lead (II) ions [210], quantitative detection of trypsin [211], or Salmonella sensing through the recognition of specific DNA fragments [212] were reported. These systems are often based on simple principles, yet providing impressive effectivity—often employing precision and selectivity of biomolecules. Pores can be incubated with a signaling molecule and then blocked inside the structure with a stimuli-responsive capping—described as molecular gate. What is more, such substrates can be calcinated and reused. There are many examples using such concept in practice. Demonstration of such system with purpose to detect *Mycoplasma fermentans* based on the DNA recognition was reported by Pla et al. [97]. The performance of the platform was also demonstrated in the breast cancer cell media. Moreover, the recycling possibility was examined via calcination at 550 °C for 5 h in order to remove the organic residues, then the substrate was successfully functionalized again. A probe enabling fast (less than 30 min) detection of *Candida albicans* was fabricated by Ribes et al. [213]. It is achieved thanks to a structure incubated with rhodamine B and the pores capped with oligonucleotide that recognize the bacteria DNA. Upon the recognition, rhodamine is released to the media. A similar arrangement was also utilized to prepare the platform designed for precise detection of cocaine with detection limit of 5 × 10^−7^ M [26]. A different example that does not fall under previous categories is the Pirani sensor [214]. The sensor is composed of a resistor on the surface of the dielectric membrane. It was prepared by deposition of thin layer of tungsten and aluminum on previously prepared p-type silicon substrate. After two-step anodization in oxalic acid, the formed tungsten oxide was selectively removed ensuring perforation at the bottom of the NAA membrane. Nickel serpentine is shaped using negative photoresist and thermal evaporation. After the resistor formation, excess material at the bottom of the membrane is released through the pores. The serpentine resistor on the top of the NAA membrane serves as an electrical heating element and temperature sensor. Below the membrane, gap spacing is provided in order to provide proper thermic isolation. Elements of the sensor and SEM images displaying produced structure are shown in Figure 16. The structure made of numerous nanopores enables lower measurable limit of pressure, due to the extent of sensing surface area and reducing the effective thermal loss. Using the membrane with porosity of 25%, it was possible to produce the device with the measurable pressure range between 0.1 mTorr to 760 Torr and was demonstrated as more sensitive than non-porous substrates. 

### 4.3. Templates 

**Figure 17:**
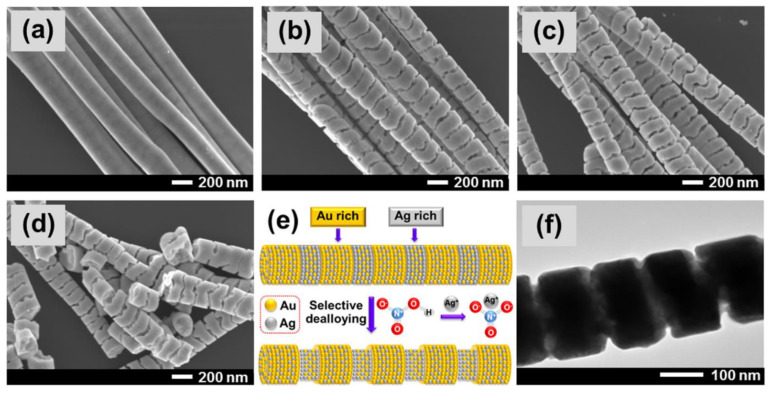
(**a**–**d**,**f**) SEM images of porous Au-Ag striped nanowires made through the interlaced electrodeposition supplemented with acid etching and (**e**) schematic of performed selective dealloying. Reprinted with permission from [223]. Copyrights 2019 Elsevier.

The use of NAA as a template has been explored for decades [47,215]. NAA template-based fabrication enables tailor-engineering of a periodic structure, as demonstrated by Lim et al. [216], where the fabrication of 2D Au nanodot single-lattices with different geometry is presented. There are many ways in which a material of interest can be introduced into NAA: Electrodeposition [217,218], salinization [206], inkjet printing [219], galvanic displacement [220], spraying [221], and polymerization [222]. With a precisely adjusted experimental setup, obtained structures feature an advanced morphology and can mix several functionalities or elements (Figure 17) [223].

Recently, a material prepared with NAA membrane as a template and coated with transition metal was proposed for spintronic applications [224]. The membrane was covered by thermal evaporation technique with layer of Ni forming antidot array on the surface. Depending on the membrane geometry, different magnetic anisotropy was obtained. The shape of NAA is also favorable for the production on nano brushes in flat and spatial (3D) configurations [10,225] as well as liberated nanowires [226,227]. However, while reports demonstrate many different combinations of materials and shape, a significant fraction remains on the concept stage. As Rath and Theato wrote in their recent review: “as presented, utilizing complex NAA architectures are still in its infancy of being used as templates for stimuli-responsive polymer. Future shows great promise for advanced applications, especially when combined with multi-responsive polymers” [228]. 

### 4.4. Membranes for Filtering and Separation

Some merits of NAA structures stimulate their use in many distinct fields. Its nanostructured array of pores is highly regular. Layers up to several micrometers thick are a desirable material to produce self-standing and durable membranes [229]. The inner side of their walls can be precisely designed to serve the application requirement [230]. Such membranes can be prepared to maintain a high level of flexibility [231,232]. With growing population and raising environmental pollution, easy-to-produce, cost-effective membranes are desirable to help us face challenges of the future. For example, NAA membranes with zinc oxide nanosheets grown on the surface were reported by Stroe et al. [233]. Their performance was demonstrated to decrease *E. coli* population by 73% in UV light over 24 h. What is more, just the pore size itself enables to separate bacteria from water [234]. 

**Figure 18:**
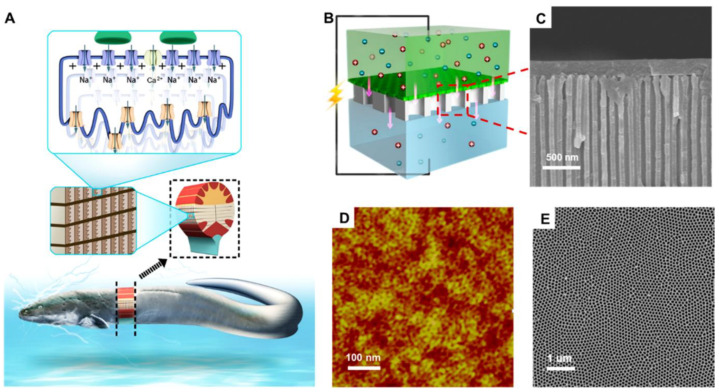
Bioinspired membrane for energy conversion. (**A**) Illustration demonstrating structure of electric eel skin with schematic display of ion channels, (**B**) schematic of bioinspired membrane for energy conversion, (**C**) SEM cross-section of the device, (**D**) AFM and (**E**) SEM top view of NAA membrane. Reprinted with permission from [241]. Copyright 2018 American Chemical Society.

NAA membranes have shown their effectivity in precise chemical filtration, for example heavy ions separation: Chemical vapor deposited carbon nanotubes inside NAA enabled removal of copper (II) and cadmium (II) [235] while decoration with Fe_3_O_4_/SiO_2_ bonded arsenic ions [236]. A different example is the pH-sensitive, smart membrane with switchable oil/water permeability functionalized with copolymer containing two blocks: pH-responsive poly(4-vinylpyridine) (P4VP) hydrophobic polystyrene (PS) [237]. Permeability of the membrane is controlled with pH that causes protonation/deprotonation of P4VP groups. When in a deprotonated state (pH neutral and alkaline), PS groups are exposed providing for hydrophobic properties of the surface—making membrane oil-permeable only. On the other hand, in an acidic environment, P4VP groups are protonated and thus hydrophilic. These membranes demonstrated performance over many cycles without decrease of the efficiency. NAA membranes are also useful for gas separation. Nanochannels sized 10-100 nm modified with a different density of octadecylphosphonic acid were demonstrated to affect permeability of the membranes [238]. Demonstration of how a thin layer of graphene oxide combined with NAA can function as a selective barrier that blocks most gases while preserving the high permeability to water vapors was reported by Petukhov et al. [239]. Selective separation can be also based on surface charge, for example charged cellulose nanofiber padding inside NAA pores enabled the selective separation of negatively charged molecules with a rejection rate close to 100% [240]. What is more, the morphology of NAA enables to reproduce more complex structures inspired by observation of nature. Inspiration with the electric eel skin morphology resulted in creation of material featuring a diode-like ion transport behavior [241]. The material is composed of two functional layers: Polymeric nanochannels with carboxyl groups additionally responsible for ion selectivity and NAA featuring hydroxyl groups and providing mechanical integrity of the membrane. Such structure demonstrated its performance in the energy conversion—energy generation is based on salinity gradient. Morphology of the electric eel skin that inspired the work, its functional principles, and architecture of the artificial counterpart are shown in Figure 18 [241]. 

### 4.5. Biological Monitoring and Cell Culture

The size of NAA structures enables unique possibility to mimic and observe biological systems at the cellular level [12,45]. NAA coated with CuO and _L_-Cys/_D_-Cys with purpose to monitor response of biological molecules was demonstrated in work of Chen et al. [242]. Furthermore, the macrophage response can be modulated with differently sized pores [243]. Nanostructural features provide the adhesive cue for macrophages affecting their shape and the spread, resulting in modified inflammatory response and osteoclastic activities. They can be also used as a model to simulate certain biological behaviors. A bioinspired nanoporous membrane as a gas exposure model mimicking the airway mechanism was reported by Jiang et al. [244]. Alveolar cells on top of the membrane were in contact with the cell culture medium at the top with exposure to the gas through the pores. The setup enabled to follow the inflammatory response of alveolar cells to the gas pollution. In different approach, researchers prepared an NAA-based platform to observe the in situ hormonal release of cultured human cells [245]. These examples reflect high compatibility of NAA with biological systems. This material can be effectively used as a surface for cell cultivation/monitoring providing superior performance [246,247]. The performance of cell cultures carried out on nanoporous anodic alumina and macroporous silicon coated with collagen and fibronectin with purpose to control the adhesion of cells to the surface was compared by Formentin et al. [248]. Properties of the fibronectin coating were superior, providing the best adhesion, morphology, and proliferation of cells. 

### 4.6. Drug Delivery

Cost-effective, biocompatible structures of NAA along with derived materials can be tailor-engineered in all dimensions. These properties stimulate the application of nanoporous anodic alumina derived materials for drug delivery. Although several approaches has been proposed involving both structural modifications [175] and stimuli-responsive release [43], instances of practical applications are few. 

One of such examples can be an implant based on the aluminum wire surface-modified through the anodization. Thin layer of NAA provided on the wire implants is examined for controlled drug delivery in vitro and ex vivo—evaluating drug release inside the bone [249]. The implant provided stable and sustained release: Sample with short pores (20 µm) reported release of 82.6% over the course of 11 days, while 45.6% of the load has been released from structures with longer pores (60 µm). Viable osteocytes in the implant surroundings detected with the bone histology demonstrating the biocompatibility of such devices. 

Furthermore, nanoparticles made of NAA—the nanotubes—were investigated for such application. A drug delivery system based on AANTs for a proof-of-concept cancer therapy was investigated [162]. The system was designed to target autophagic and endoplasmic reticulum stress—nanotubes were loaded with thapsigargin (TG). Modified AANTs were initially examined with regards to their impact on the cell function, then examined with the human breast cancer cells. AANTs induced cellular response and were successfully internalized, demonstrating possibility of intracellular drug delivery. Furthermore, 3-methyladenine was used as an autophagy inhibitor that was demonstrated to improve cancer cell killing effect of TG-loaded nanotubes [162]. These nanotubes were later examined in vivo on the murine model (Balb/c mice, eight weeks) and introduced by intravenous injection and subcutaneous implantation routes [163]. The intravenous path did not display any impact on the viability while low and moderate doses were applied, with higher doses resulting in detectable accumulation in liver and spleen. More pronounced effects were observed with subcutaneous route that triggered inflammatory response. 

In a recent review, drug delivery perspectives, providing an update to the state of the art of NAA and NAA-derived materials in the field were discussed by Kapruwan et al. [48]. 

### 4.7. Functional Layer for Composites

**Figure 19:**
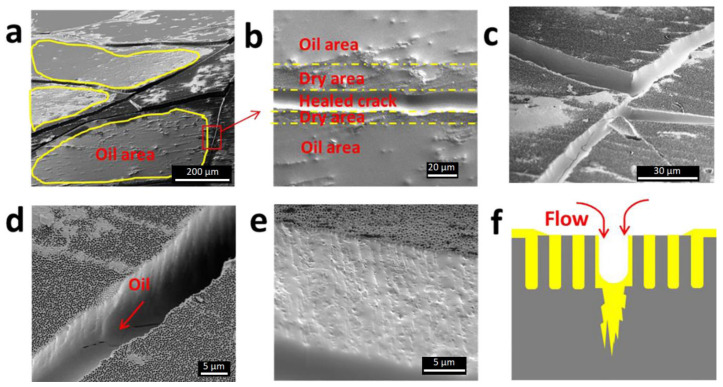
Cryo-SEM images of (**a**–**c**) generated micro-cracks, (**d**) surface surrounding the crack, (**e**) cross-section of lubricant filled crack, and (**f**) schematic of repair process. Reprinted with permission from [253]. Copyright 2019 Elsevier.

Since nanoporous anodic alumina can be grown into aluminum and its alloys, even whole components can be enhanced with an additional functionality. A coverage of aluminum surface with an NAA coating is a common practice that greatly improves the corrosion resistance and mechanical properties of the material. Apart from chemical resistance, the hard NAA layer boost surface hardness and tribological durability [80,250]. The improvement of the thermal management provided through the NAA coating may serve as another benefit. NAA layers can significantly reduce (~14%) the thermal resistance and junction temperature of the surface—which could be beneficial for packaging and casing design [251]. NAA membranes have shown passive cooling effect that resulted in 2.6 °C below ambient temperature of the cooler when exposed to direct sunlight [252]. Complex morphology of the NAA facilitates for preparation of composites. A method to completely fill alumina nanopores of 50 µm long with mineral oil by means of vacuum impregnation was shown by Wu et al. [253]. Such a combination exhibits self-healing properties over the wear damage and surface cracks additionally providing corrosion protection over extended time periods. Morphology of the structure, behavior around the cracks, and schematic of repair behavior is shown in Figure 19 [253]. In a different example, preparation of composite made of Ni nanopillars embedded inside the NAA structure through electrodeposition was reported by Tishkovich et al. [254]. Layers of electrodeposited Ni are commonly utilized to improve mechanical and corrosion properties of many metals. The combination with NAA was demonstrated to exhibit higher corrosion resistance in saline aqueous solution as compared to bulk Ni layer.

The aforementioned examples are not an exhaustive list covering discoveries of recent decades. Instead, the aim is to provide an update with the most recent contributions in the field and a context to account for the exponential growth of publications involving nanoporous anodic alumina. The scope involves an insight into the fabrication mechanism and design tools with intention to provide synthesis of a comprehensive knowledge about the material. Our intention is to disseminate NAA fabrication as a nanotechnology tool that—while not completely unique—definitely possess a few advantages, and that sometimes, it is imagination that restricts innovation.

## 5. Conclusions

The anodization of aluminum has changed greatly since its discovery almost 100 years ago. Decades of work done by many researchers—step by step—unveiled subtleties of the process slowly turning robust industrial method employed to cover machine parts with the protective layer, into a sophisticated and precise nanotool. Applications of NAA vary greatly, from cost-effective templates, smart surfaces to biomaterials and sensing platforms—providing vast utility. Precise control of geometrical features known today was possible due to exploration of the formation mechanism, close analysis with advanced equipment at nano- and atomic scale during different stages of formation process sometimes coincidental results and definitely, a continuous dialogue between groups of researchers. Compared to the past, today’s aluminum anodization is a mature technology—facile, cost-effective, and environmentally friendly—yet another promise of a better future. We understand more and have accurate control over the material formation. NAA can be designed for specific interaction with light due to tailored morphology, choice of electrolyte can enhance photoluminescence or improve conductivity, while smart combination of simple techniques can turn amorphous oxide into chemically resistant and hard corundum, simultaneously preserving its porous morphology. 

Exploration of possibilities related with NAA does not slow down. Photonic crystals itself or filled with responsive medium are still in the development and may revolutionize sensing, and microelectronics. The ability to precisely tailor nanoparticles is useful, but definitely restricted on the side of interactions with biological systems. Significant improvement has been done in taming technology to provide highly ordered surfaces even using low-grade aluminum substrates. This may enable to universalize its utility as a functional coating. Many applications have been already implemented as a proof-of-concept. It can be expected that in the future, these novelties will be introduced into already existing systems, sometimes as a fast and easy approach to enhance properties of already utilized material, but also as precisely engineered materials capable of competing and replacing more expensive alternatives. 

## Figures and Tables

**Table 2:** Recently established applications of NAA-based structures.

Application	Reported Utility	Refs
Photonic structures	Reviews on photonic structures	[34,46,186]
	Principles of photonic crystals	[187,188,189]
	Fabry-Perót interferometer	[190]
	Gradient-index filters	[191]
	Distributed Bragg reflectors	[192]
	Optical bandpass filters	[193]
	Human protein heavy ions real-time interaction monitoring	[194]
	Tailoring of optical properties with pulse anodization	[149]
	Design of phase shift defect in effective refractive index	[195]
	Hybrid distributed DBRs and apodized GIF photonic structure	[196]
	Characterization of thermotropic ferroelectric liquid crystals confined in the NAA	[197,198,199]
	Glass transition of discotic liquid crystals in one-dimensional fluid	[200]
	Adjustable optical anisotropy by self-assembly of liquid crystals confined in porous structure	[201]
Sensors	NAA template-assisted fabrication of chromium substrate for SERS detection of heavy ions in aqueous solutions	[202]
	Au NPs array on NAA for SERS detection of hemoglobin	[203]
	Controlled fabrication of periodic plasmonic dimer arrays for SERS	[204]
	Graphene-NAA composite for SERS sensing	[205]
	Label-free optical sensor based on interferometric reflectance spectroscopy for TNF-α detection	[206]
	NAA-based interferometer for copper sensing	[207]
		[208]
	Rhodamine B release triggered by Staphylococcus aureus detection	[209]
	Determination of Pb^2+^ with DNAzyme	[210]
	Reflectance spectroscopy based biosensor for determination of trypsin	[211]
	Salmonella sensing through DNA sequence recognition	[212]
	Reusable, molecular gated-NAA for detection of *Mycoplasma fermentans*	[97]
	Molecular gated-NAA for detection of *Candida albicans*	[213]
	Molecular gated-NAA for sensitive detection of cocaine	[26]
	Adjustable sensor based on the metallic resistor suspended on NAA membrane	[214]
Templates	Reviews of NAA template-assisted fabrication	[47,215]
	Fabrication of 2D Au nanodot arrays with tailorable geometric features for photocatalysis enhancement	[216]
	Nanostructured surface for photocatalysis	[217]
	Fabrication of Bi NWs for microelectronics	[218]
	Perowskite NWs with a tunable emission wavelength	[219]
	Electrodes for the electrochemical denitrification	[220]
	Metal-free coating for the broadband infrared absorption	[221]
	Polymer-brush structure confined in the NAA	[222]
	Segment Au-Ag nanowires for SERS detection	[223]
	Fabrication of Ni antidot arrays for spinotronic applications	[224]
	Review on NAA-derived electrochemical energy storage devices	[10]
	Co magnetic NWs	[225]
	Fe-Pd NWs for magnetic/catalytic spinoelectronics	[226]
	Ca_2_FeIn Hensler alloy NWs for spintronics application	[227]
	Review on NAA-molded stimuli-responsive polymer structures	[228]
Membranes for filtering and separation	Tailor-engineering of narrow pore NAA membranes	[229]
	Growing covalent organic frameworks on porous substrates for molecule-sieving membranes with pores tunable from ultra- to nanofiltration	[230]
	Mechanical properties of NAA membranes	[231,232]
	Photocatalytic membrane for water disinfection	[233]
	Steric-based removal of Coliform bacteria	[234]
	Removal of Cu^2+^ and Cd^2+^ with pH controlled permeability	[235]
	Removal of As from aqueous media	[236]
	Switchable hydrophobicity of the membrane for selective oil/water emulsion separation	[237]
	Efficient separation of hydrocarbons	[238]
	Gas dehumidification method with selective water vapor-permeable membrane	[239]
	Selective rejection of polar molecules	[240]
	The electric eel inspired structure for energy conversion	[241]
Biological monitoring and cell culture	Reviews on NAA-based biosensors	[12,45]
	Biomimetic nanochannels for enhanced biomolecule response	[242]
	Modulation of osteo-immune response of macrophages	[243]
	Structure with three-phase interface for gas exposure as a lung airway model	[244]
	NAA-based substrate for in situ monitoring of hormonal release from human cell culture	[245]
	Osteogenic differentiation induced with NAA morphology	[246]
	Pore size-dependent growth of N2a cells	[247]
	Control of cell adhesion with functional coating of collagen and fibronectin	[248]
Drug delivery	Impact of NAA pore geometry on DOX sustained-release profile	[175]
	pH sensitive NAA platform for sustained drug release	[43]
	Bone ex vivo evaluation of drug release from NAA surface-modified aluminum wire implants	[249]
	Proof-of-concept cancer therapy with nanotube-based drug delivery system targeting autophagic and endoplasmic reticulum stress	[162]
	*In vivo* nanotubes nanotoxicity study on murine model	[163]
	Drug delivery perspectives of NAA-derived materials review	[48]
Functional layer for composites	Enhanced wear resistance through multiphase lubrication mechanism	[250]
	Tartaric-sulfuric acid NAA as “green” alternative for chromic-NAA protective layers	[80]
	NAA coating for reduction of thermal resistance and junction temperature	[251]
	Daytime passive radiative cooling layer	[252]
	Lubricant infused structure with self-healing properties and enhanced corrosion resistance	[253]
	NAA casing to reduce Ni corrosion in brine	[254]

## Data Availability

Data sharing is not applicable to this article as no new data were created or analyzed in this study.
